# A Novel SINE Transposable Element-Associated lncRNA Regulates Zygotic Genome Activation in Porcine Parthenogenetically Activated Embryos

**DOI:** 10.3390/cells15181707

**Published:** 2026-09-19

**Authors:** Tianyao He, Shanlong Zhang, Shichao Zhang, Dongsong Liu, Han Li, Jun Song, Zhonghua Liu, Jun-Xue Jin, Jing-Tao Sun

**Affiliations:** Key Laboratory of Animal Cellular and Genetics Engineering of Heilongjiang Province, College of Life Science, Northeast Agricultural University, Harbin 150030, China; hetianyao@neau.edu.cn (T.H.); 15698220028@163.com (S.Z.); 15846386919@163.com (S.Z.); liuds1007@163.com (D.L.); lylh0095@163.com (H.L.); songjun@neau.edu.cn (J.S.)

**Keywords:** SINE-associated lncRNA, lncRNA, ZGA, embryo development, *EP300*

## Abstract

Zygotic genome activation (ZGA) is a pivotal event during early mammalian embryonic development and is regulated by multiple molecular factors. In mice, previous studies have demonstrated that long non-coding RNAs (lncRNAs) and transposable elements (TEs) participate in the regulation of this process. However, the functional roles of TEs-associated lncRNAs in porcine ZGA remain largely unknown. In this study, we identified a previously unannotated lncRNA associated with a SINE transposable element sequence in porcine parthenogenetically activated embryos, which we named *LincSAKP*. *LincSAKP* exhibited peak expression at the 8-cell stage, coinciding with ZGA, was predominantly localized in the nucleus, and lacked protein-coding potential. Functional analyses demonstrated that depletion of *LincSAKP* resulted in developmental arrest at the 8-cell stage, accompanied by reduced chromatin accessibility, impaired nascent transcription, and aberrant expression of multiple ZGA marker genes and transcription factors. Transcriptomic analysis combined with rescue experiments identified *EP300* as a critical downstream target of *LincSAKP*. Notably, Depletion of either *LincSAKP* or *EP300* impairs histone H3K27ac modification. *EP300* overexpression effectively alleviated the developmental defects caused by *LincSAKP* depletion. Collectively, our findings identify *EP300* as a downstream mediator of *LincSAKP* in regulating zygotic genome activation, providing insights into the functional network of SINE-associated lncRNAs during parthenogenetically activated early embryonic development. This study expands our understanding of the functional roles of TE-associated non-coding RNAs in early embryonic development and provides a potential theoretical basis for improving the efficiency of in vitro culture of porcine embryos.

## 1. Introduction

Zygotic genome activation (ZGA), also called Embryonic genome activation (EGA) is a critical developmental transition during embryogenesis, referring to the process by which the embryonic genome switches from a transcriptionally silent state to widespread transcriptional activation. It also marks the fundamental transition of developmental control from maternally derived factors to the embryonic genome itself [1]. ZGA is regulated by multiple factors, including epigenetic reprogramming, chromatin remodeling, transcription factors, and translational regulation [1,2,3]. In addition, long non-coding RNAs (lncRNAs) are important regulators of gene expression during ZGA. Several non-coding RNAs, such as microRNAs (miRNAs) and lncRNAs, can regulate the expression of key ZGA genes through interactions with mRNAs [4,5]. However, the mechanisms by which lncRNAs regulate ZGA remain unclear, and their roles during porcine embryonic development have been rarely investigated. Therefore, further exploration of the regulatory mechanisms of lncRNAs in porcine ZGA is of great importance.

Long non-coding RNAs (lncRNAs) play important roles in gene regulation and epigenetic processes during early mammalian embryonic development. For example, *Xist* mediates X chromosome inactivation [6,7,8], *Air* and *Kcnq1ot1* mediate genomic imprinting [9,10,11], *PancIl17d* regulates DNA demethylation to influence embryonic development [12], and *LincGET* mediates the first cell fate decision [13,14,15]. In addition, lncRNAs also regulate the process of ZGA. Using single-cell RNA sequencing, Zhang et al. identified a large number of lncRNAs and found that many lncRNAs expressed during the cleavage stage (the ZGA stage) were associated with cell cycle regulation, transcription, translation, and oxidative phosphorylation [16]. Therefore, investigating the regulatory mechanisms of lncRNAs will facilitate a better understanding of the molecular basis of embryonic development during in vitro culture and provide a theoretical basis for improving the treatment of reproductive disorders and the efficiency of in vitro embryo culture.

Transposable elements (TEs) are mobile DNA sequences that constitute approximately 50% of mammalian genomes and were once referred to as “jumping genes” or “selfish DNA” [17,18]. Based on their transposition mechanisms, TEs are classified into DNA transposons, which rely on transposases, and retrotransposons, which transpose through RNA intermediates. Retrotransposons include long terminal repeat (LTR) elements, such as human endogenous retroviruses (HERVs), and non-LTR elements, including long interspersed nuclear elements (LINEs) and short interspersed nuclear elements (SINEs), the latter of which are major drivers of genome evolution [19]. During early mammalian embryonic development, TEs are highly activated and transcribed during zygotic genome activation (ZGA) and regulate developmental processes through multiple mechanisms [20]. At the cis-regulatory level, TEs can function as enhancers, promoters, or three-dimensional (3D) genome regulatory elements to directly regulate the expression of neighboring genes. For example, the primate-specific LTR5Hs acts as an enhancer in primordial germ cells and is bound by SOX17 and NANOG to regulate genes involved in germline development. In mice, the LTR sequences of *MuERVL*, such as *MT2_Mm*, possess promoter activity and drive the expression of hundreds of ZGA-associated genes [21,22]. Taking advantage of this property, Macfarlan et al. established a *MERVL* reporter system and subsequently identified “2-cell-like cells” (2CLCs) with totipotent features, providing an important in vitro model for ZGA research [20]. The pioneer factor NR5A2 promotes chromatin opening by binding to SINE B1/Alu elements located near key ZGA genes at the mouse 2-cell stage, thereby regulating ZGA and the transition of embryos from totipotency to pluripotency [23,24]. In addition, TEs regulate chromatin structure and function by altering epigenetic modifications. *MuERVL* promotes ZGA by increasing histone acetylation and enhancing chromatin plasticity [25,26,27], whereas the timely activation and silencing of *LINE1* regulate global chromatin accessibility during early embryonic development [28]. Collectively, these studies demonstrate the diverse roles of TEs in the regulation of ZGA and provide important insights into the molecular basis of early embryonic development.

In addition to cis-regulation and epigenetic regulation, TEs can also exert trans-regulatory functions through the formation of chimeric RNA transcripts [29,30]. Studies have shown that TEs can serve as sequence sources or regulatory modules of long non-coding RNAs (lncRNAs), thereby participating in the fine regulation of ZGA. For example, the human ERV subfamily *MLT2A1* generates a large number of diverse chimeric RNAs through fusion with downstream transposable elements, including LINE1 and Alu. The conserved 5′ region of these transcripts binds to HNRNPU and recruits RNA polymerase II, whereas the diverse 3′ fusion sequences expand their genomic targeting range, thereby forming a mutually reinforcing regulatory network that drives global transcriptional activation of ZGA genes [31]. In mice, LincGET, which originates from an ERV-derived chimeric locus, has been shown to be indispensable for cleavage of 2-cell embryos. Depletion of *LincGET* results in embryonic arrest at the G2 phase of the 2-cell stage, whereas its asymmetric expression during the 2- to 4-cell stages promotes blastomere differentiation toward the inner cell mass (ICM) fate. Mechanistically, *LincGET* interacts with CARM1 to promote H3R26me2 deposition, thereby activating the expression of ICM-associated genes [13,14,15]. In addition, Sakashita, Kitano, and colleagues systematically demonstrated through siRNA, antisense oligonucleotide (ASO), CRISPR interference (CRISPRi), and mRNA rescue experiments that transcription-associated MERVL RNA is essential for ZGA [20]. Similarly, the timely presence of LINE1 RNA is also indispensable for ZGA and the regulation of global chromatin accessibility [28]. In pigs, the SINE-associated lncRNA *SAWPA* promotes JNK transcription through its SINE sequence, thereby regulating the process of ZGA [32]. These findings collectively demonstrate the importance of TE-associated lncRNAs in the regulation of early embryonic development. Given that approximately 80% of lncRNAs contain TE-derived sequences [33], the identification of TE-associated lncRNAs and elucidation of their regulatory mechanisms on target genes will become an important direction for future ZGA research. With the rapid development of long-read sequencing, low-input multi-omics technologies, advanced in vitro models, and precise genome-editing technologies, studies on the functions of TE-associated lncRNAs during early embryonic development are expected to achieve new breakthroughs.

At present, the regulatory mechanisms of TEs during early porcine embryonic development remain largely unclear. In particular, whether TE-associated lncRNAs act as key regulators during ZGA in porcine embryos has yet to be determined. Therefore, the identification and functional characterization of TE-associated lncRNAs are of critical importance. Another important question is the identity of the target genes regulated by TE-associated, especially SINE-associated, lncRNAs during ZGA, which also warrants further investigation and validation. Based on our previous screening of SINE-associated lncRNAs, we analyzed their spatiotemporal expression patterns during porcine embryonic development and identified a lncRNA that was highly expressed during ZGA. This lncRNA is located between the *KTN1* and *PELI2* genes. Characterization of its biological properties demonstrated that it is a SINE-associated lncRNA consisting of two exons, with a length of 2832 nt, predominantly localized in the nucleus. We therefore named it *LincSAKP*. Functional analyses showed that depletion of *LincSAKP* resulted in developmental arrest at the 8-cell stage, reduced chromatin accessibility during ZGA, and decreased levels of nascent RNA transcription. In addition, the expression of key ZGA-associated genes and transcription factors was markedly altered. Mechanistically, RNA sequencing revealed that *LincSAKP* depletion affected the expression of a large number of transcription factors, among which *EP300* was identified as a key downstream target. Our findings support a model in which *LincSAKP* contributes to ZGA, at least in part, through *EP300*-associated regulation of H3K27ac. Overexpression of *EP300* rescued the developmental arrest caused by *LincSAKP* depletion. These findings indicate that *LincSAKP* plays an important regulatory role during ZGA in parthenogenetically activated porcine embryonic development and provide a theoretical basis for improving reproductive medicine and the efficiency of in vitro embryo culture.

## 2. Materials and Methods

### 2.1. Primer Design

Primers were designed using Primer Premier 5 software (Table S1) and synthesized by Beijing Genomics Institute (Beijing, China).

### 2.2. Antibodies

The following antibody was used for immunofluorescence (IF) analysis: anti-BrdU antibody (sheep polyclonal, #ab1893; Abcam, Cambridge, UK), diluted 1:100 in blocking buffer for the detection of BrdU incorporation during DNA replication. H3K27ac antibody (rabbit polyclonal, #ab4729; Abcam), diluted 1:50 in blocking buffer, for the detection of H3K27ac in embryos. EP300 antibody (rabbit polyclonal, #PA1-848; Thermo, Waltham, MA, USA), diluted 1:50 in blocking buffer, for the detection of EP300 in embryos. All secondary antibodies (Alexa Fluor^®^ 555-conjugated goat anti-rabbit IgG, #ab150078; Abcam, and Alexa Fluor^®^ 555-conjugated anti-sheep IgG; #ab150178; Abcam) were diluted 1:500 in blocking buffer.

### 2.3. Embryo Culture and Collection

Porcine ovaries were collected from a local slaughterhouse and transported to the laboratory in a thermos container containing physiological saline maintained at 37 °C. Upon arrival, ovaries were repeatedly washed with 37 °C physiological saline supplemented with penicillin (S9137; Sigma, St. Louis, MO, USA) and streptomycin (P3032; Sigma). Cumulus–oocyte complexes (COCs) were aspirated from 3 to 6 mm follicles using a 10 mL syringe equipped with an 18-gauge needle. The collected COCs were washed three times with HEPES-buffered medium (H3784-1KG; Sigma). COCs with evenly distributed cytoplasm and compact cumulus cell layers consisting of at least three layers were selected for in vitro maturation. Approximately 50 COCs were cultured in 500 μL of TCM-199 medium (3100-027; Invitrogen, Carlsbad, CA, USA) supplemented with 0.14% polyvinyl alcohol (PVA; P8136; Sigma), 10 ng/mL epidermal growth factor (EGF; E4127; Sigma), 0.57 mM cysteine (C7602; Sigma), 0.5 IU/mL pregnant mare serum gonadotropin (PMSG; hor272; PROSPEC, Rehovot, Israel), and 0.5 IU/mL human chorionic gonadotropin (hCG; hor250; PROSPEC). COCs were cultured at 39 °C in a humidified atmosphere containing 5% CO_2_ for 42–44 h to complete in vitro maturation. Following maturation, COCs were transferred into Eppendorf tubes (MCT-150-C; AXYGEN, Union City, NJ, USA) containing 0.1% hyaluronidase and repeatedly pipetted to remove surrounding cumulus cells. Mature oocytes with an extruded first polar body were selected for parthenogenetic activation. Subsequently, the oocytes were equilibrated for 5 min in activation medium containing 0.2 mM CaCl_2_ and 50 mM mannitol. Two direct current pulses (1.0 kV/cm, 30 μs, 0.5 s interval) were then applied using an electrofusion apparatus (CF-150/B, BTX, Holliston, MA, USA). The activated embryos were washed three times with PZM-3 medium and transferred to PZM-3 pre-equilibrated at 39 °C for further culture.

Activated embryos were cultured in PZM-3 medium consisting of 10 mL PZM-3 basal medium (final concentrations in basal medium: 108 mM NaCl, 10 mM KCl, 0.35 mM KH_2_PO_4_, 0.5 mM MgSO_4_·7H_2_O, 25 mM NaHCO_3_, 0.2 mM sodium pyruvate, 2 mM calcium lactate, 1 mM L-glutamine, and 5 mM hypotaurine) supplemented with BSA to a final concentration of 0.15% (*w*/*v*), 200 μL BME amino acid solution (B6766; Merck, Darmstadt, Germany), and 100 μL MEM non-essential amino acid solution (M7145; Merck), resulting in a final culture volume of 10,800 μL. Embryos were collected at 12, 24, 48, 72, 96, 108, and 168 h after PA, corresponding to the 1-cell, 2-cell, 4-cell, 8-cell, day 4.0 morula (4.0D MO), day 4.5 morula (4.5D MO), and blastocyst (BL) stages, respectively. Embryos at the corresponding morphological stages were collected at different time points.

### 2.4. Quantitative Real-Time PCR

Total RNA was extracted from embryos using TRIzol™ Reagent (15596026; Invitrogen). After removal of residual medium, embryos were lysed in 100 μL TRIzol and stored at −80 °C. Samples were thawed at 4 °C, supplemented with additional TRIzol to a final volume of 400 μL, vortexed, and incubated at room temperature for 5 min to dissociate nucleoprotein complexes. Samples were vigorously shaken for 2 min, incubated at room temperature for 2–3 min, and centrifuged at 12,000× *g* for 15 min at 4 °C. The aqueous phase (200 μL) was transferred to a new tube, mixed with 400 μL isopropanol and 2 μL glycogen, and precipitated at −20 °C for 1 h. Samples were centrifuged at 12,000× *g* for 15 min at 4 °C, and the supernatant was removed. RNA pellets were washed with 400 μL of 75% ethanol, centrifuged at 7500× *g* for 5 min at 4 °C, and the washing step was repeated once. After complete removal of residual ethanol and air drying at room temperature, RNA was dissolved in RNase-free water preheated to 60 °C.

Reverse transcription was performed using the RevertAid™ First Strand cDNA Synthesis Kit (M1632; Thermo, Waltham, USA). The reverse transcription reaction contained 4 μL enzyme mix, 20 μL RT buffer mix, 2 μL RNA, and 14 μL RNase-free water. The reaction was performed at 42 °C for 1 h followed by 95 °C for 15 min.

Quantitative PCR was performed using TB Green Premix Ex Taq (RR420A; Takara, Shiga, Japan). The reaction mixture contained 10.0 μL TB Green, 0.40 μL ROX II, 1.00 μL cDNA, 0.50 μL each of forward and reverse primers, and 7.60 μL nuclease-free water. Reactions were performed on an ABI 7500 Fast Real-Time PCR System. Porcine 18S rRNA was used as the internal control, and relative expression levels were calculated using the 2^^−ΔΔCt^ method. Regarding the primers, Primer amplification efficiencies were validated by standard curves using 5-fold serial dilutions of pooled cDNA, with efficiencies ranging from 90% to 110% (R^2^ > 0.99). Each sample was run in triplicate technical replicates, and Cq values with SD > 0.3 were excluded. No-template controls (NTC) and no-reverse-transcription controls (NRT) were included in each run to exclude contamination and genomic DNA carryover, respectively. Amplicon specificity was confirmed by melt-curve analysis (65 °C to 97 °C). 18S rRNA was selected as the reference gene based on previous validation studies demonstrating its stable expression across porcine preimplantation developmental stages and under different experimental conditions [34].

### 2.5. Subcellular Localization Analysis

To determine the subcellular localization of *LincSAKP* in embryos, subcellular fractionation analysis was performed. A total of 100 8-cell stage embryos were lysed in pre-chilled RLN1 buffer (50 mM Tris-HCl, pH 8.0, 140 mM NaCl, 1.5 mM MgCl_2_, 0.5% NP-40, and 2 mM Vanadyl Ribonucleoside Complex) on ice for 5 min. Samples were centrifuged at 300× *g* for 2 min at 4 °C. The supernatant containing the cytoplasmic fraction was transferred into a DEPC-treated tube and stored at 4 °C. The remaining pellet was resuspended in pre-chilled RLN2 buffer (50 mM Tris-HCl, pH 8.0, 500 mM NaCl, 1.5 mM MgCl_2_, 0.5% NP-40, and 2 mM Vanadyl Ribonucleoside Complex), incubated on ice for 5 min, and centrifuged at 16,360× *g* for 2 min at 4 °C. The supernatant containing the nucleoplasmic fraction was transferred into a new DEPC-treated tube and stored on ice, while the remaining pellet was collected as the chromatin fraction. Total RNA from each fraction was extracted using 400 μL TRIzol reagent and subsequently subjected to reverse transcription and quantitative real-time PCR (RT-qPCR) analysis.

### 2.6. Microinjection

To investigate the regulatory relationship and biological functions of *LincSAKP* and *EP300*, embryos were microinjected 6 h after PA. Embryos were placed in MAN buffer and microinjected on the stage of a Nikon inverted microscope. RNA ASOs, negative control ASOs, and in vitro-transcribed *LincSAKP* and *EP300* mRNAs were individually injected using a FemtoJet microinjection system (Eppendorf, Hamburg, Germany). The injection parameters were set as follows: injection pressure, 150 hPa; compensation pressure, 50 hPa; injection time, 0.7 s; and injection volume, approximately 10 pL per embryo. For in vitro transcription of mRNAs, target gene fragments containing the T7 promoter sequence were amplified by PCR and transcribed using the HiScribe™ T7 High Yield RNA Synthesis Kit (E2040S, NEB, Ipswich, MA, USA). Capped mRNAs were generated by co-transcriptional addition of Cap1 analog (NEB, S1404) and polyadenylated with E. coli Poly(A) Polymerase (NEB, M0276) at 37 °C for 30 min to add ~150–200 nt poly(A) tails. The synthesized RNA products were purified by LiCl precipitation and subsequently used for microinjection.

### 2.7. 5′ and 3′ Rapid Amplification of cDNA Ends (RACE)

To determine the full-length transcript sequence of *LincSAKP*, 5′ and 3′ rapid amplification of cDNA ends (RACE) were performed using the 5′/3′ RACE Kit 2nd Generation (03353621001; Roche, Basel, Switzerland) and the 3′-Full RACE Core Set with PrimeScript™ RTase (6106; Takara, Shiga, Japan), respectively. Gene-specific primer sequences are listed in Table S1.

For 5′ RACE, total RNA was extracted from 200 8-cell stage embryos using the TRIzol method. Reverse transcription was performed using the gene-specific primer *LincSAKP*-5′RACE-R1 under the following conditions: 55 °C for 60 min and 85 °C for 5 min. The purified cDNA was subjected to terminal transferase-mediated poly(A) tail addition to generate dA-tailed cDNA. Two rounds of nested PCR were performed. The first PCR used dA-tailed cDNA as the template with *LincSAKP*-5′RACE-R2 and Oligo dT-Anchor Primer. The second PCR used the first-round PCR product as the template with *LincSAKP*-5′RACE-R3 and PCR Anchor Primer. Final PCR products were analyzed by electrophoresis and confirmed by sequencing.

For 3′ RACE, total RNA was extracted from 200 8-cell stage embryos and reverse transcribed using the 3′ RACE Adaptor under the following conditions: 42 °C for 60 min and 70 °C for 15 min. Nested PCR was subsequently performed. The first PCR used *LincSAKP*-3′RACE-F1 and Gene Specific Outer Primer, while the second PCR used the first-round PCR product as template with *LincSAKP*-3′RACE-F2 and Gene Specific Inner Primer. The final products were analyzed by electrophoresis and verified by sequencing.

### 2.8. BrdU Staining

At 6 h after PA, embryos were injected with *LincSAKP* ASO or negative control NC ASO and cultured in PZM-3 medium for an additional 56 h. BrdU was added to the culture medium at a final concentration of 20 μg/mL, and aphidicolin was simultaneously added at a final concentration of 0.5 μg/mL as a negative control. At 72 h after PA, the zona pellucida was removed using acidic Tyrode’s solution (pH 2.5). Embryos were immediately washed three times with MAN solution and fixed with 4% paraformaldehyde at room temperature for 30 min. After two washes with washing buffer (5 min each), embryos were permeabilized for 30 min and blocked with blocking buffer for 1 h at room temperature. Embryos were incubated overnight at 4 °C with primary antibody diluted 1:100 in blocking buffer. After washing, embryos were incubated with fluorescent secondary antibody diluted 1:500 in blocking buffer for 2 h at room temperature in the dark. Following secondary antibody incubation, embryos were washed three times (5 min each), stained with Hoechst 33342 (1:1000 dilution in washing buffer) for 10 min in the dark, washed three additional times, mounted, and analyzed by fluorescence microscopy. BrdU fluorescence intensity was quantified for all measurable nuclei within each embryo. The nuclear fluorescence measurements were then averaged within each embryo to obtain a single embryo-level value, and each embryo was treated as one independent statistical unit. Quantification and statistical analyses were performed as described in Section 2.15 and Section 2.17.

### 2.9. EU Staining

To assess nascent RNA synthesis, embryos injected with NC ASO or *LincSAKP* ASO after PA were cultured in PZM-3 medium for 64 h. EU was then added to the culture medium at a final concentration of 10 μM, and embryos were cultured for an additional 8 h. After removal of the zona pellucida, embryos were fixed with 4% paraformaldehyde and washed twice with washing buffer (5 min each). Embryos were subsequently permeabilized with permeabilization buffer for 30 min at room temperature and washed once. Embryos were incubated with freshly prepared Click-iT^®^ reaction mixture for 30 min in the dark, followed by sequential washing with reaction rinse buffer and washing buffer. Nuclear staining was performed using Hoechst 33342. After washing, embryos were mounted and subjected to fluorescence imaging analysis. EU fluorescence intensity was quantified over the entire embryo, and one mean fluorescence-intensity value was obtained for each embryo and used as a single independent statistical observation. Embryos were obtained from three independent embryo-culture batches, and statistical analysis of the embryo-level fluorescence measurements was performed as described in Section 2.17. An α-amanitin-treated group was included as a transcription-inhibition control for the EU-labeling assay (Figure S3B).

### 2.10. In Vivo DNase I-TUNEL Assay

Embryos injected with RNA fragments and cultured for 72 h until the 8-cell stage were collected and washed with PBS prior to analysis. Extraction buffer A (50 mmol/L NaCl, 3 mmol/L MgCl_2_, 0.5% Triton X-100, 300 mmol/L sucrose, and 25 mmol/L HEPES) and extraction buffer B (identical composition without Triton X-100) were prepared. Embryos were permeabilized in extraction buffer A on ice for 5 min and subsequently washed with extraction buffer B. The embryos were then incubated in extraction buffer B containing 1 U/mL DNase I for 5 min. After DNase I treatment, embryos were fixed with fixation solution for 30 min. TdT enzyme and fluorescein-dUTP labeling solution were mixed at a ratio of 1:10, and embryos were incubated with the mixture at 37 °C in the dark for 1 h. Embryos were then washed three times with washing buffer (5 min each). Nuclear staining was performed using Hoechst 33342 (1:1000 dilution in washing buffer) for 10 min in the dark, followed by three additional washes (5 min each). Finally, embryos were mounted on glass slides and subjected to fluorescence microscopy imaging. For quantification, the fluorescence intensity and nuclear diameter of all measurable nuclei within each embryo were averaged to obtain one embryo-level value, and each embryo was treated as an independent statistical unit. Embryos were obtained from three independent embryo-culture batches, with both treatment groups represented in each batch. The total number of embryos and their distribution among batches are reported in Figure 3’s legend. Statistical analyses of embryo-level measurements were performed as described in Section 2.17. A no-DNase control and a TUNEL baseline control were included to assess assay specificity and background fluorescence, respectively (Figure S3A).

### 2.11. RNA Interference and Embryo Developmental Analysis

To investigate the function of *LincSAKP* and *EP300* during early embryonic development, antisense oligonucleotides (ASOs) were designed and synthesized. Embryos at the pronuclear stage were microinjected with *LincSAKP* ASOs, *EP300* ASOs, or corresponding negative control ASOs. All ASOs, including the negative control, were chemically modified with 2′-O-methyl (2′-OMe) and a full phosphorothioate (PS) backbone to enhance nuclease resistance and binding affinity, and were microinjected using ASO solutions at a concentration of 10 μM, with the negative control sequence 5′-CGTACTCGCGATGCGTACTC-3′ (confirmed by BLAST v2.17.0 to have no known homology to the pig genome). Following injection, embryos were cultured in PZM-3 medium, and developmental progression was monitored. The cleavage rate was evaluated at 48 h after parthenogenetic activation, and blastocyst formation was assessed at 168 h after activation. Embryos were collected at different developmental stages for subsequent molecular analyses. The efficiency of depletion was determined by RT-qPCR.

### 2.12. RNA-Seq Library Preparation and Transcriptome Analysis

Approximately 400 eight-cell-stage embryos were pooled for each RNA-seq library. Three independent biological replicates were generated for each treatment group (NC ASO and *LincSAKP* ASO-1), resulting in three libraries per group and six libraries in total. Thus, approximately 1200 embryos were used per group and approximately 2400 embryos were used in total for RNA extraction and library construction.

RNA-seq libraries were constructed using the NEBNext^®^ Ultra™ II RNA Library Prep Kit for Illumina (NEB, #E7770) following the manufacturer’s protocol, with poly(A) mRNA enrichment using the NEBNext Poly(A) mRNA Magnetic Isolation Module (NEB, #E7490). Libraries were quantified and validated for fragment size distribution using a Qubit^®^ 4.0 Fluorometer (Thermo Fisher Scientific, Waltham, MA, USA) and an Agilent 2100 Bioanalyzer (Agilent Technologies, Santa Clara, CA, USA). Paired-end sequencing (150 bp) was performed on the MGISEQ-2000 platform, generating approximately 20–25 million raw reads per sample. Raw sequencing reads were processed by removing adapter sequences and low-quality reads using Trimmomatic (v0.39). Clean reads were aligned to the pig reference genome (Sus scrofa 11.1, Ensembl release 104) using HISAT2 (v2.2.1). Gene expression levels were quantified using featureCounts (v2.0.1). Differential-expression analysis between the NC ASO and *LincSAKP* ASO-1 groups was performed using DESeq2 (v1.34.0) based on gene-level read counts. Genes with a nominal *p* value ≤ 0.05 and |log2 fold change| ≥ 1 were retained as candidate differentially expressed genes for downstream exploratory analyses. Functional enrichment analysis, including KEGG pathway analysis, was performed to identify biological pathways associated with DEGs.

### 2.13. Plasmid Construction and Overexpression Experiments

The full-length sequences of *LincSAKP* and *EP300* were amplified by PCR and cloned into expression vectors. For overexpression experiments, in vitro-transcribed *LincSAKP* and *EP300* mRNAs were prepared as described above and microinjected into embryos. To perform rescue experiments, embryos were co-injected with *LincSAKP* ASO and *EP300* mRNA or with *EP300* ASO and *LincSAKP* mRNA. In vitro-transcribed LincSAKP and EP300 mRNAs (full-length sequences, without exogenous 5′/3′ UTRs) were microinjected at 100 ng/μL. Embryonic development was subsequently evaluated at 168 h after parthenogenetic activation, and blastocyst formation rates were calculated.

### 2.14. Open Reading Frame (ORF) Prediction and Protein-Coding Potential Analysis

The potential protein-coding ability of *LincSAKP* was evaluated using the NCBI ORF Finder tool. Predicted ORFs were analyzed using NCBI BLASTp to identify potential conserved protein domains. To experimentally validate the coding potential of predicted ORFs, selected ORF sequences (ORF7, ORF20, ORF15, and ORF4) were cloned upstream of an EGFP reporter lacking the ATG start codon to generate fusion constructs. The constructs were expressed in 8-cell stage embryos and HEK293T cells, and fluorescence signals were examined to determine whether *LincSAKP*-derived ORFs possessed protein-coding activity.

### 2.15. Immunofluorescence Staining

Embryos were fixed with 4% paraformaldehyde at room temperature for 30 min after removal of the zona pellucida. After washing, embryos were permeabilized and blocked before incubation with primary antibodies at 4 °C overnight. After washing, embryos were incubated with corresponding fluorescent secondary antibodies for 2 h at room temperature in the dark. Nuclei were stained with Hoechst 33342, and embryos were mounted for fluorescence microscopy analysis. Fluorescence intensity was quantified using ImageJ 1.46r software. For immunofluorescence quantification, each embryo was treated as an independent statistical unit. The mean fluorescence intensity of all measurable nuclei within each embryo was calculated and used as one embryo-level observation. Biological replicates were defined as independent embryo-culture experiments using different batches of ovaries. The total number of embryos and their distribution among biological batches are reported in the corresponding figure legends, and statistical analyses were performed as described in Section 2.17.

### 2.16. RNA FISH

After removal of the zona pellucida from 8-cell-stage embryos using acidic operating solution (10 µL HCl in 1 mL MAN), embryos from different developmental stages were sequentially washed in three PBS droplets containing 6 mg/mL BSA. The embryos were then transferred onto Superfrost/Plus microscope slides (12-550-15, Fisher, Pittsburgh, PA, USA) and allowed to dry as quickly as possible. Subsequently, the embryos were fixed and permeabilized with 4% paraformaldehyde (PFA). The subcellular localization of the lncRNA in embryos was detected using the D-T-G LncRNA In Situ Hybridization Kit (D-074, FOCO, Guangzhou, China) according to the following procedure: 100 µL of Solution A was applied to the samples, followed by incubation at 37 °C for 20 min, after which Solution A was discarded. This step was repeated sequentially with 100 µL of Solution B and 100 µL of Solution C under the same conditions. The samples were then washed with 1× PBS for 5 min, and the PBS was discarded. After a brief rinse with anhydrous ethanol, 100 µL of hybridization buffer was added, and pre-hybridization was performed at 55 °C for 1 h. Two *LincSAKP*-targeting RNA probes were generated by in vitro transcription using T7 RNA Polymerase (NEB, M0251S) with 555-UTP (Thermo, A32762) incorporated as the fluorescent label; the corresponding probe-template primer sets (FISH-*LincSAKP*-1 and FISH-*LincSAKP*-2) are listed in Table S1. The probes were used at 10 μg/mL hybridization solution. The probe was diluted 1:10 in hybridization buffer, heated to eliminate secondary structures, and applied to the slides. Hybridization was carried out overnight at 85 °C for 14 h in the dark. Following five washes with wash buffer, nuclei were counterstained, and the slides were mounted for imaging. This procedure was performed to investigate the localization of *LincSAKP*.

### 2.17. Statistical Analyses

All data are presented as mean ± SEM from at least three independent biological replicates, with each biological replicate defined as an independent embryo-culture experiment using a different batch of ovaries. For developmental outcomes, including blastocyst formation, cleavage, and developmental arrest at specific stages, the numbers of embryos with and without each outcome were analyzed as binomial data. Generalized linear mixed-effects models (GLMMs) with a binomial distribution and logit link function were fitted, with Treatment included as a fixed effect and experimental Batch included as a random intercept to account for batch-to-batch variation. Pairwise comparisons among treatment groups were performed based on contrasts from the fitted GLMMs. All tests were two-sided, and *p* < 0.05 was considered statistically significant. Exact *p* values and model-based 95% confidence intervals are provided in the corresponding Supplementary Figures and Tables.

For fluorescence-based assays, each embryo was treated as an independent statistical unit. For assays in which fluorescence was quantified at the nuclear level, measurements from all measurable nuclei within an embryo were averaged to obtain one embryo-level value. For whole-embryo fluorescence assays such as EU labeling, the mean fluorescence intensity of the entire embryo was directly used as the embryo-level observation. To account for batch-to-batch variation, embryo-level fluorescence data were analyzed using an additive two-way ANOVA model with Treatment and Batch included as fixed main effects (Treatment + Batch). No Treatment × Batch interaction term was fitted because of the limited number of embryo-level observations within individual batches.

For qPCR data, technical replicates were averaged within each biological replicate, and statistical inference was performed on ΔCt values obtained from three independent biological replicates. Relative expression levels calculated using the 2^^−ΔΔCt^ method were used for graphical presentation. Because control and treatment samples were generated in parallel within the same experimental batches, two-sided paired t-tests were used for comparisons between the two groups, with experimental batch serving as the pairing factor. Given the limited number of biological replicates (*n* = 3), normality was not formally tested. *p* < 0.05 was considered statistically significant.

## 3. Results

### 3.1. LincSAKP Is a SINE-Associated, 8-Cell Stage-Enriched, and Nuclear-Localized lncRNA

**Figure 1:**
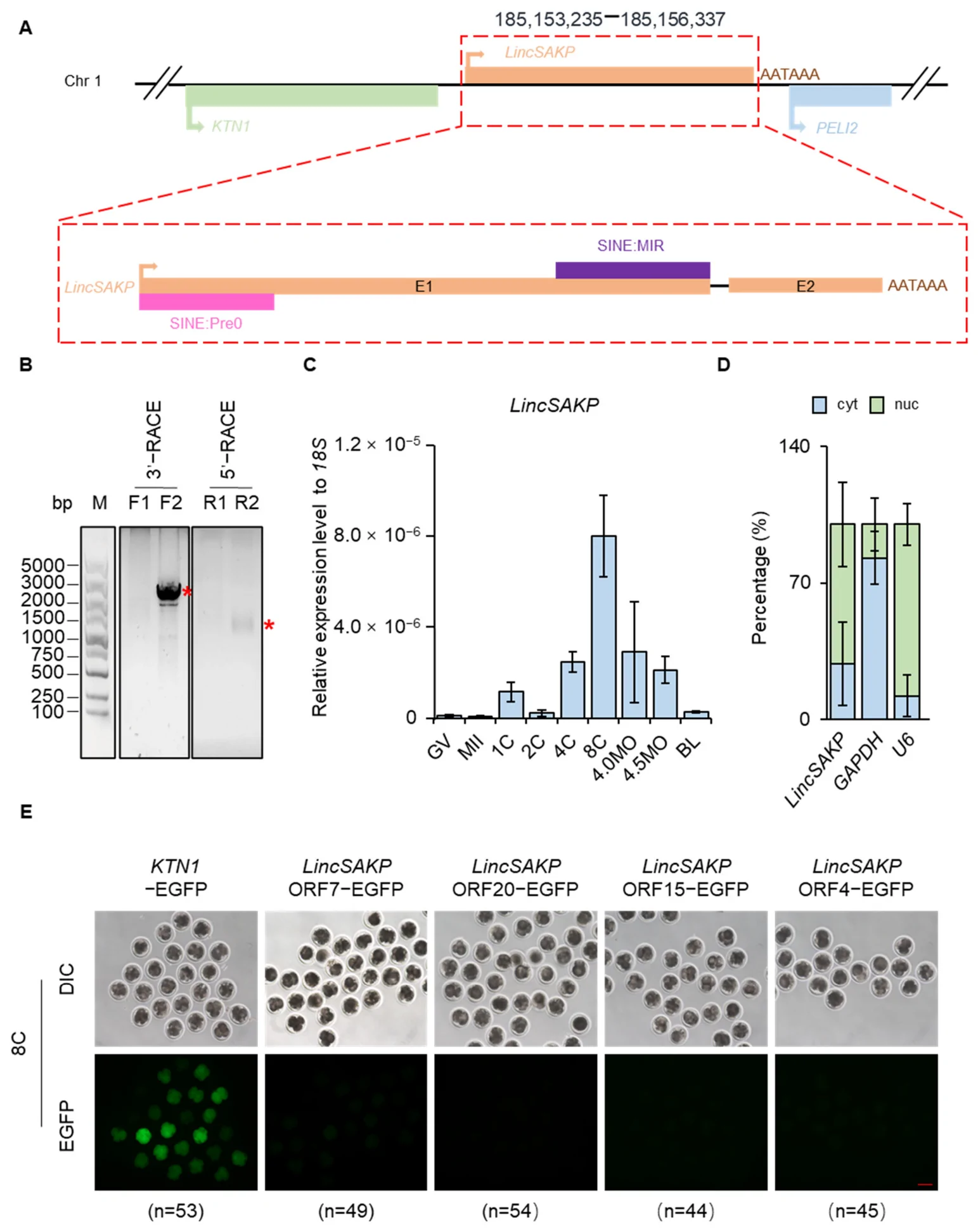
*LincSAKP* is a SINE-related lncRNA located between the *KTN1* and *PELI2* genes. (**A**) Genomic sequence information of *LincSAKP*. *LincSAKP* is a transcript containing a SINE sequence, located between the *KTN1* and *PELI2* genes, composed of two exons, with a length of 2832 nt, and containing an AATAAA polyadenylation signal. *LincSAKP* is located on Chr1: 185,153,235–185,156,337 of the pig reference genome Sus scrofa 11.1. (**B**) 3′ RACE and 5′ RACE results for *LincSAKP*. Gene-specific primers (F1, F2, R1 and R2) are shown in Supporting Information Figure S1A. * Indicates the bands corresponding to the correct bands of 3′ RACE and 5′ RACE for *LincSAKP*. Approximately 200 early 8-cell embryos were used for each RACE experiment, and three experimental replicates were performed. (**C**) RNA expression pattern of *LincSAKP* during porcine early embryonic development from oocyte to blastocyst, determined by qPCR. GV, germinal vesicle oocyte; 1C, 1-cell stage; 2C, 2-cell stage; 4C, 4-cell stage; 8C, 8-cell stage; 4D, 4-day morula; 4.5D, 4.5-day morula; BL, 7-day blastocyst. Error bars represent S.E.M. Approximately 50 embryos of each stage were used, and three experimental replicates were performed. (**D**) Subcellular localization of *LincSAKP* using RNA fractionation and qPCR analysis. *LincSAKP* was detected in both the nuclear and cytoplasmic fractions, with predominant nuclear enrichment. Error bars represent S.E.M. Nuc, nucleoplasm; Cyt, cytoplasm. GAPDH and U6 serve as cytoplasmic and nuclear controls, respectively. About 100 early 8-cell embryos were used for each experiment, and three experimental replicates were performed. (**E**) Assessment of the protein-coding potential of *LincSAKP* by removing the ATG start codon and fusing with EGFP. The ORF represents the predicted region with possible protein-coding capacity. Supporting information is shown in Figure S1A. Scale bar, 100 μm. Three experimental replicates were performed.

SINE-associated long non-coding RNAs (lncRNAs) are multifunctional regulatory molecules that participate in key developmental processes, including embryonic development, neurogenesis, and myogenesis, by regulating transcription, translation, and RNA stability [30,35]. However, whether SINE-associated lncRNAs regulate zygotic genome activation (ZGA) during early porcine embryonic development remains unclear. A previous study demonstrated that the SINE-associated lncRNA *SAWPA* plays an important regulatory role during ZGA in porcine early embryos. In addition, our previous study identified 14 SINE-associated lncRNAs [36]. To identify SINE-associated lncRNAs potentially involved in ZGA, we analyzed the spatiotemporal expression patterns of these candidate lncRNAs during early parthenogenetically activated porcine embryonic development, selected those with high expression during ZGA, and characterized their biological properties. Based on these analyses, we identified a SINE-associated lncRNA containing MIR and Pre0 elements located between the *KTN1* and *PELI2* genes. We named this lncRNA *LincSAKP* (long intergenic non-coding RNA associated with SINE, *KTN1*, and *PELI2*) (Figure 1A and Figure S1).

As the previously identified *LincSAKP* sequence did not represent the full-length transcript, we performed 3′ and 5′ rapid amplification of cDNA ends (RACE) using gene-specific primers to obtain the complete transcript sequence and genomic locus of *LincSAKP*. The sequencing results were analyzed using the UCSC BLAT tool. We found that *LincSAKP* is located between the *KTN1* and *PELI2* genes and has a single transcript isoform consisting of two exons. The transcript contains the SINE elements MIR and Pre0, a canonical polyadenylation signal (AATAAA), and is 2832 nt in length (Figure 1A,B and Figure S1A).

To further investigate whether *LincSAKP* is associated with ZGA, we examined its expression pattern in parthenogenetic embryos from the germinal vesicle (GV) stage to the day 7 blastocyst (BL) stage by quantitative real-time PCR (RT–qPCR). *LincSAKP* was expressed at low levels from the GV to the 4-cell stage, reached its highest expression level at the 8-cell stage, and gradually declined thereafter until the blastocyst stage (Figure 1C). The peak expression of *LincSAKP* coincided with the timing of ZGA, suggesting that it may play a regulatory role during this process.

As the coding potential of *LincSAKP* was unknown, we next characterized its subcellular localization. Nuclear and cytoplasmic fractions were isolated from 8-cell embryos and analyzed by RT–qPCR. Compared with the positive controls, U6 (nuclear) and GAPDH (cytoplasmic), *LincSAKP* was predominantly localized in the nucleus, consistent with the nuclear enrichment observed for many functional lncRNAs [14], but this localization alone does not establish non-coding status (Figure 1D). We further analyzed the open reading frames (ORFs) of *LincSAKP* using the NCBI ORF Finder. Although multiple short ORFs were identified and BLASTP analysis suggested the presence of conserved protein domains, no Kozak sequence was detected, indicating limited protein-coding potential (Figure S1B). To further evaluate its coding capacity, the four longest predicted ORFs (ORF7, ORF20, ORF15, and ORF4) were individually fused to an EGFP expression vector lacking the ATG initiation codon and expressed in both 8-cell embryos and HEK293T cells. Fluorescence analysis showed that none of these ORFs exhibited protein-coding activity in either 8-cell embryos or HEK293T cells (Figure 1E and Figure S1C). Collectively, these findings demonstrate that *LincSAKP* is a non-coding RNA.

### 3.2. Interference with LincSAKP Causes Embryonic Arrest at the 8-Cell Stage and Impairs Embryonic Development

**Figure 2:**
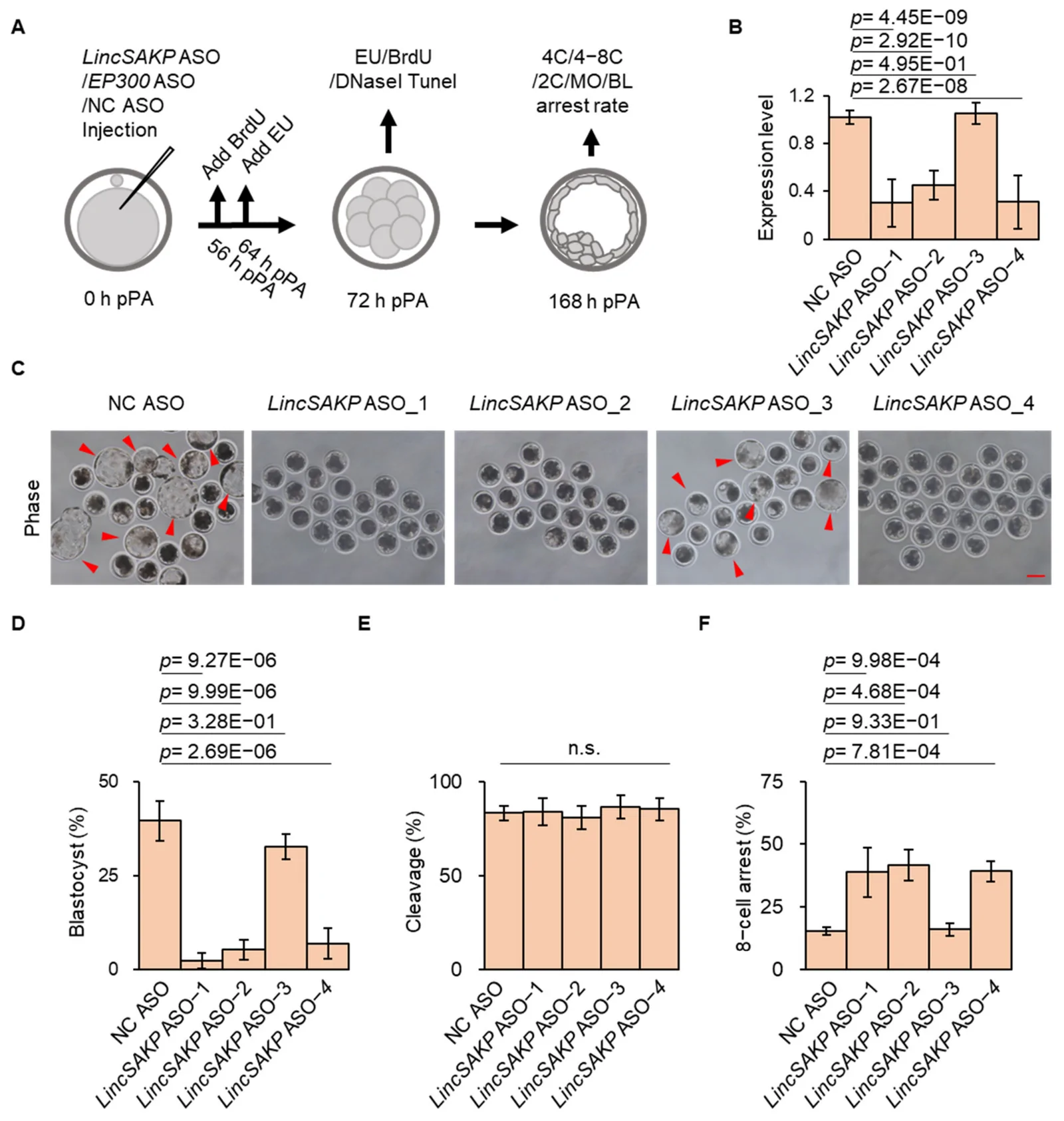
Depletion of *LincSAKP* causes embryonic arrest at the 8-cell stage. (**A**) Schematic diagram of developmental monitoring and statistical analysis. (**B**) Efficiency analysis of *LincSAKP* depletion. *LincSAKP* ASOs 1–4 were injected at 6 h post-parthenogenetic activation (pPA), and embryos were collected at 72 h pPA (8-cell stage) for qPCR analysis. Error bars represent S.E.M. Approximately 50 embryos of each stage were used. 18S served as an internal reference gene. At least three experimental replicates were performed for each RNAi injection. (**C**) Loss of *LincSAKP* leads to embryonic developmental arrest at the 8-cell stage. Photographs were taken at 168 h pPA (blastocyst stage). Scale bar, 100 μm. At least three experimental replicates were performed for each RNAi injection. (**D**) Loss of *LincSAKP* impairs blastocyst formation; the blastocyst rate of embryos injected with *LincSAKP* ASOs was significantly decreased. At least three experimental replicates were performed for each RNAi injection. (**E**) Loss of *LincSAKP* does not affect embryo cleavage. After injection of *LincSAKP* ASOs at 6 h pPA, the number of cleaved embryos was counted at 48 h of development, and the cleavage rate was calculated; there was no significant difference in the cleavage rate between ASO-injected embryos and controls. At least three experimental replicates were performed for each RNAi injection. (**F**) Loss of *LincSAKP* causes 8-cell arrest; the 8-cell arrest rate of embryos injected with *LincSAKP* ASOs was significantly increased. At least three experimental replicates were performed for each RNAi injection.

To investigate the role of *LincSAKP* during early porcine embryonic development, we performed RNA interference (RNAi)-based depletion and evaluated embryonic developmental competence. Because *LincSAKP* is predominantly localized in the nucleus, antisense oligonucleotides (ASOs) were used to silence its expression. ASOs were microinjected into embryos at the pronuclear stage, and blastocyst formation rates were assessed at 168 h after parthenogenetic activation (PA) to compare embryonic development between the *LincSAKP* ASO and negative control (NC) ASO groups (Figure 2A). A total of four ASOs targeting *LincSAKP* (ASO-1, ASO-2, ASO-3, and ASO-4) were designed and evaluated. Embryos were collected at the 8-cell stage after microinjection, and depletion efficiency was determined by RT–qPCR. Compared with the NC ASO group, *LincSAKP* ASO-1, ASO-2, and ASO-4 achieved depletion efficiencies of approximately 80% (Figure 2B). Correspondingly, embryos in these groups exhibited developmental arrest, accompanied by a significant reduction in blastocyst formation at 168 h (Figure 2C). We further quantified embryonic development in each group and found that depletion of *LincSAKP* by ASO-1, ASO-2, or ASO-4 significantly reduced the blastocyst formation rate compared with the control group (Figure 2D and Figure S2A, Table 1 and Table S2). To exclude the potential effects of microinjection, cleavage rates were assessed at 48 h after PA. No significant differences in cleavage rates were observed among the *LincSAKP* ASO-1, ASO-2, ASO-4, and NC ASO groups (Figure 2E and Figure S2A, Table 1 and Table S2). This indicated that the injection procedure did not affect embryonic development.

In early porcine embryos, ZGA occurs during the 4-cell to 8-cell transition [36]. To better assess whether gene depletion affects ZGA, we included both the 4- to 8-cell arrest rate (the sum of embryos arrested at either the 4-cell or 8-cell stag) and the 8-cell arrest rate (the proportion of embryos arrested at the 8-cell stage) as complementary indicators, allowing a more comprehensive evaluation of ZGA impairment. Our analysis revealed that embryos injected with *LincSAKP* ASO-1, ASO-2, or ASO-4 showed a markedly higher proportion of developmental arrest during the transition from the 4-cell to the 8-cell stage than control embryos. Approximately 60% of embryos arrested between the 4-cell and 8-cell stages, whereas nearly 40% arrested at the 8-cell stage (Figure 2F and Figure S2A, Table 1 and Table S2). Given that the 8-cell stage corresponds to the major ZGA stage in porcine embryos, these results suggest that *LincSAKP* depletion impairs ZGA during early embryonic development.

### 3.3. Interference with LincSAKP Affects Zygotic Genome Activation

To confirm that the developmental phenotypes observed upon *LincSAKP* depletion were specifically attributable to the loss of *LincSAKP* rather than off-target effects of the ASO, we constructed an ASO-resistant *LincSAKP* mutant (*LincSAKP*-mut) by introducing nucleotide substitutions within the ASO-1-binding region (Figure S2B). The wild-type and mutant ASO-1-binding sequences, including the nucleotide substitutions, are provided in Figure S2B. Co-injection of LincSAKP mut mRNA with *LincSAKP* ASO-1 significantly rescued the developmental arrest, restoring blastocyst formation and reducing 8-cell arrest rates without affecting cleavage (Figure S2C–E), confirming the specificity of the knockdown phenotype. To further assess *LincSAKP* expression under the rescue condition, we examined embryos co-injected with *LincSAKP* ASO-1 and *LincSAKP* mut. RNA-FISH showed a stronger *LincSAKP* signal with predominant nuclear enrichment in the rescue group (Figure S2F), and RT-qPCR confirmed a significant increase in total LincSAKP RNA abundance compared with the *LincSAKP* ASO-1 group (Figure S2G). As these assays detect total *LincSAKP* rather than specifically distinguishing the endogenous and ASO-resistant transcripts, the results were interpreted together with the developmental rescue data. Collectively, these findings support successful expression of the rescue construct and the specificity of the developmental phenotype caused by *LincSAKP* ASO-1 treatment.

**Figure 3:**
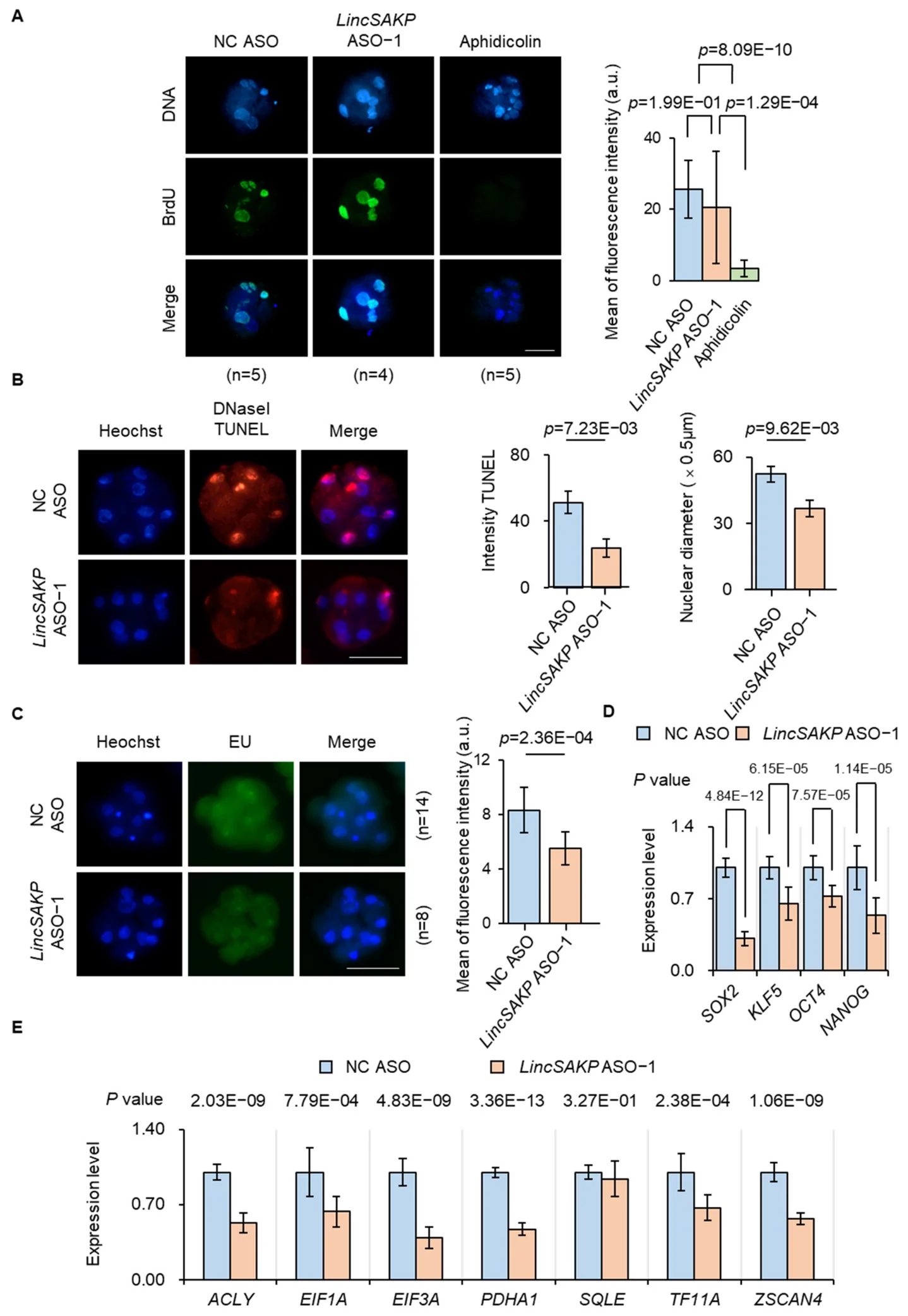
*LincSAKP* depletion results in developmental arrest at the 8-cell stage with effects on ZGA initiation. (**A**) Depletion of *LincSAKP* does not affect DNA integrity and replication. We used BrdU to visualize S and G2 phases. Aphidicolin-treated embryos were arrested at the S phase without DNA replication. There was no difference in fluorescence intensity between NC ASO and *LincSAKP* ASO-1. Embryos were collected at pPA 72 h (8-cell stage) for immunofluorescence analysis. Scale bar, 100 μm. The NC ASO, *LincSAKP* ASO-1, and aphidicolin groups contained 3, 4, and 5 embryos, respectively, distributed across three independent biological batches as 1/1/1, 2/1/1, and 2/1/2. All three treatment groups were represented in every biological batch. Data are shown as mean ± SEM. (**B**) *LincSAKP* ASO-1-mediated depletion was associated with reduced DNase I–TUNEL fluorescence intensity and nuclear diameter in 8-cell-stage embryos. Scale bar, 100 μm. NC ASO and *LincSAKP* ASO-1 groups contained 7 and 11 embryos, respectively, distributed across three independent biological batches as 2/2/3 and 3/3/5. Both treatment groups were represented in every batch. Data are shown as mean ± SEM. The no-DNase and TUNEL baseline controls are shown in Figure S3A. (**C**) The nascent RNA transcription during ZGA was detected by EU labeling after *LincSAKP*-ASO-1 depletion. EU was added to the culture medium at pPA 64 h, and EU signals were detected at pPA 72 h. Scale bar, 100 μm. EU fluorescence was quantified over the entire embryo, and each embryo was treated as one independent statistical unit. NC ASO and LincSAKP ASO-1 groups contained 14 and 8 embryos, respectively, distributed across three independent biological batches as 4/5/5 and 2/3/3. (**D**) Expression of genes related to pluripotency, such as *SOX2*, *KLF5*, *OCT4* and *NANOG*. Compared with NC ASO, the expression levels of these genes in *LincSAKP* ASO-1-depleted embryos were decreased. Embryos injected with ASO were collected at pPA 72 h (8-cell stage) for qPCR analysis. Error bars represent S.E.M. Approximately 100 embryos were used for each group, and three experimental replicates were used. (**E**) Expression of genes related to major ZGA initiation, such as *ACLY*, *EIF1A*, *EIF3A*, *PDHA1*, *SQLE*, *TFIIA* and *ZSCAN4*, in comparison between NC ASO and *LincSAKP* ASO-1. Embryos injected with ASO were collected at pPA 72 h at the 8-cell stage for qPCR analysis. Error bars represent S.E.M. Approximately 100 embryos were used for each group, and three experimental replicates were used. 18S served as an internal reference gene.

Having established the specificity of LincSAKP knockdown using the ASO-resistant rescue experiment, we next investigated whether *LincSAKP* regulates ZGA progression. Since *LincSAKP* ASO-1 exhibited the highest knockdown efficiency among the tested ASOs, it was selected for all subsequent mechanistic experiments. To evaluate whether *LincSAKP* depletion affects DNA replication, BrdU incorporation was detected by immunofluorescence staining. Embryos injected with *LincSAKP* ASO-1 or NC ASO were cultured until 56 h after PA, followed by BrdU treatment, and immunofluorescence analysis was performed at 72 h. An aphidicolin-treated group, in which DNA replication was blocked, was included as a negative control (Figure 3A). The results showed that BrdU fluorescence intensity was not significantly different between *LincSAKP* ASO-1 and NC ASO embryos, indicating that *LincSAKP* depletion did not affect DNA replication (Figure 3A).

Global chromatin accessibility is an important factor influencing ZGA. To determine whether *LincSAKP* regulates ZGA activation by affecting chromatin accessibility, we performed an in vivo DNase I–TUNEL assay to assess global chromatin openness. Embryos were injected with *LincSAKP* ASO or NC ASO at the 1-cell stage and cultured until 72 h for DNase I–TUNEL analysis. Global chromatin accessibility was evaluated by comparing nuclear fluorescence intensity and nuclear diameter. The results showed that embryos injected with *LincSAKP* ASO exhibited significantly lower TUNEL fluorescence intensity than control embryos (Figure 3B, and Figure S3A). Furthermore, we measured nuclear diameter as an additional parameter associated with chromatin organization [14]. Consistent with the DNase I-TUNEL results, embryos injected with *LincSAKP* ASO exhibited a significantly reduced nuclear diameter compared with controls (Figure 3B). These results show that *LincSAKP* ASO-1-mediated depletion is associated with reduced global chromatin accessibility at the 8-cell stage.

To determine whether *LincSAKP* depletion affects the initiation of major ZGA at the 8-cell stage, we used 5-ethynyl uridine (EU) staining to measure nascent RNA transcription. Embryos injected with *LincSAKP* ASO-1 or NC ASO were cultured until 64 h after PA, followed by EU labeling, and immunofluorescence analysis was performed at 72 h (Figure 2A). The results showed a significant difference in EU signals between NC ASO and *LincSAKP* ASO embryos at the 8-cell stage, with *LincSAKP* ASO embryos exhibiting lower fluorescence intensity than NC ASO embryos (Figure 3C and Figure S3B). These results show that *LincSAKP* ASO-1-mediated depletion is associated with reduced nascent transcription in 8-cell-stage embryos.

ZGA is essential for the maternal-to-zygotic transition and contributes to the establishment of the totipotent state [37,38]. Because transcription factors play important roles in ZGA regulation, we next examined the expression of the pluripotency-associated genes *SOX2*, *KLF5*, *OCT4*, and *NANOG* by RT-qPCR. Based on paired comparisons of batch-level ΔCt values, *SOX2*, *KLF5*, and *NANOG* were significantly reduced following *LincSAKP* ASO-1 treatment, whereas *OCT4* showed a similar downward trend that did not reach statistical significance (Figure 3D). In addition, several genes, including *ACLY*, *EIF1A*, *EIF3A*, *PDHA1*, *SQLE*, *TFIIA*, and *ZSCAN4*, have been identified as ZGA marker genes [39,40,41,42]. We further analyzed the expression levels of ZGA marker genes in 8-cell stage embryos from the *LincSAKP* ASO and NC ASO groups by RT–qPCR. *LincSAKP* ASO-1 treatment was associated with significant reductions in *ACLY*, *EIF1A*, *EIF3A*, *PDHA1*, *TFIIA* and *ZSCAN4*, while *SQLE* remained unchanged (Figure 3E). Together with the reduced chromatin accessibility and nascent transcription observed above, these expression changes are consistent with impaired ZGA progression following *LincSAKP* ASO-1-mediated depletion.

### 3.4. LincSAKP Regulates ZGA Progression by Modulating the Expression of the ZGA-Associated Transcription Coactivator Factor EP300

lncRNAs commonly regulate various biological processes by modulating the expression of upstream or downstream genes. To further investigate the molecular mechanism by which *LincSAKP* regulates ZGA during early porcine embryonic development, we first examined the expression pattern of the neighboring gene *KTN1*, which is located near *LincSAKP*. We found that *KTN1* exhibited the highest expression level at the 1-cell stage and gradually decreased during embryonic development, showing an expression pattern similar to maternal factors, which differed from the expression pattern of *LincSAKP* (Figure S4A). Furthermore, we examined the expression levels of *KTN1* and *PELI2* in 8-cell embryos injected with *LincSAKP* ASO-1. Compared with the NC ASO group, depletion of *LincSAKP* did not affect the expression levels of *KTN1* or *PELI2* (Figure S4B). These results indicate that *LincSAKP* may not regulate ZGA progression through modulation of its neighboring genes.

**Figure 4:**
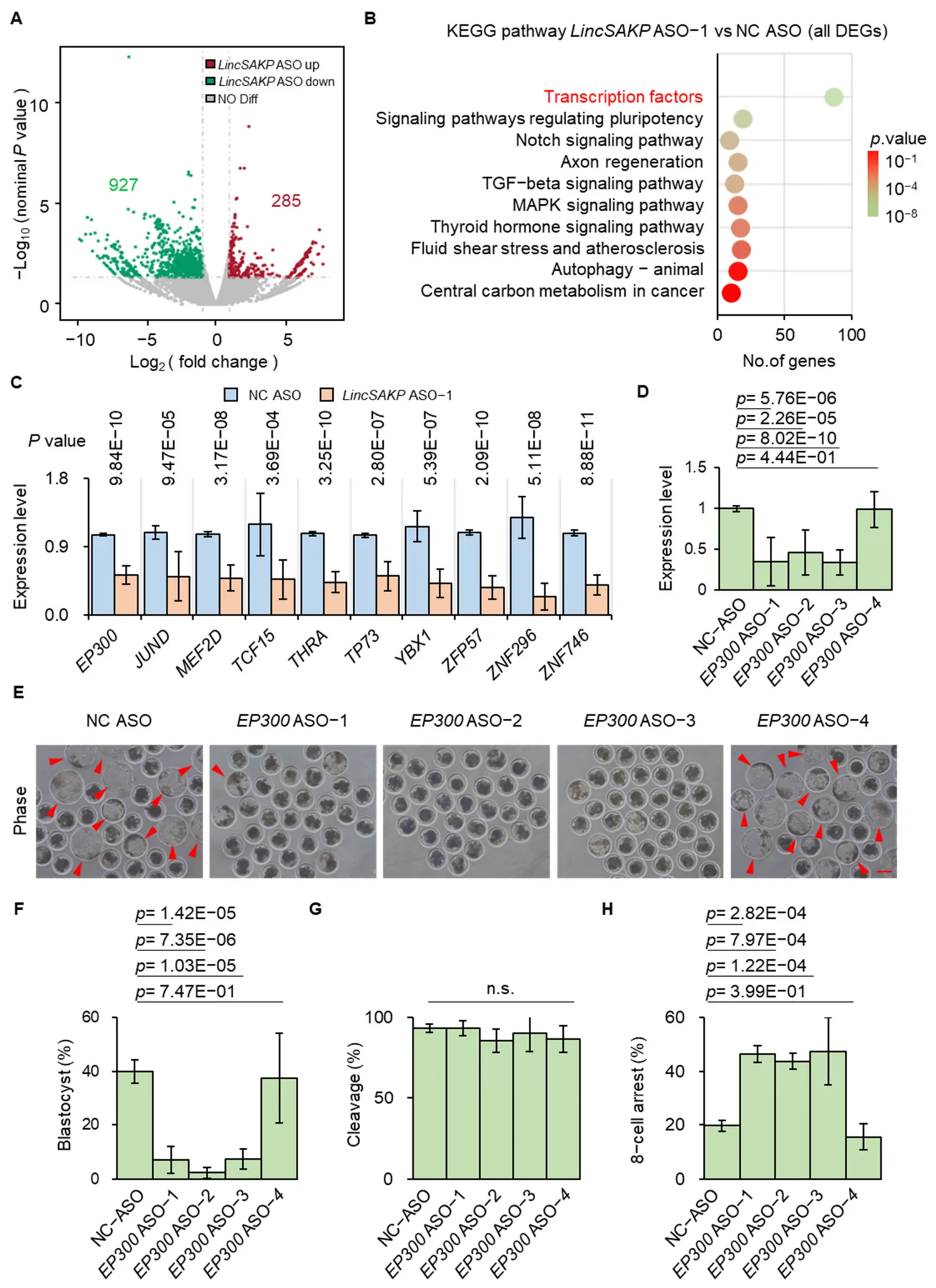
Depletion of *LincSAKP* downregulates *EP300* expression, and *EP300* depletion impairs embryonic development. (**A**) Volcano plot of candidate differentially expressed genes identified by RNA-seq between NC ASO and *LincSAKP* ASO-1-treated 8-cell embryos. Using nominal *p* ≤ 0.05 and |log2 fold change| ≥ 1 as exploratory screening criteria, 927 genes were downregulated and 285 genes were upregulated. Approximately 400 embryos were pooled for each RNA-seq library, with three independent biological replicates per group (six libraries in total). (**B**) KEGG pathway analysis of DEGs showed that transcription factors were mainly affected by *LincSAKP* ASO-1 depletion. (**C**) The expression of genes related to transcription factors and transcriptional coactivator was significantly affected by *LincSAKP* ASO-1 depletion. Error bars represent S.E.M. Approximately 100 embryos were used for each group, and three experimental replicates were performed. (**D**) Depletion efficiency analysis of *EP300*. *EP300* ASOs 1–4 were injected at 6 h post-parthenogenetic activation (pPA), and embryos were collected at pPA 72 h (8-cell stage) for qPCR analysis. Error bars represent S.E.M. Approximately 50 embryos of each stage were used. 18S served as an internal reference gene. At least three experimental replicates were performed for each RNAi injection. (**E**) Depletion of *EP300* leads to embryonic arrest at the 8-cell stage. Photographs were taken at pPA 168 h (blastocyst stage). Scale bar, 100 μm. At least three experimental replicates were performed for each RNAi injection. (**F**) Depletion of *EP300* impairs blastocyst formation; the blastocyst rate of embryos injected with *EP300* ASOs was significantly decreased. At least three experimental replicates were performed for each RNAi injection. (**G**) Depletion of *EP300* does not affect embryo cleavage. After injection of *EP300* ASOs at 6 h pPA, the number of cleaved embryos was counted at 48 h of development, and the cleavage rate was calculated; there was no significant difference in the cleavage rate between ASO-injected embryos and controls. At least three experimental replicates were performed for each RNAi injection. (**H**) Depletion of *EP300* causes 8-cell arrest; the 8-cell arrest rate of embryos injected with *EP300* ASOs was significantly increased. At least three experimental replicates were performed for each RNAi injection.

To further characterize the molecular changes associated with embryonic arrest following *LincSAKP* ASO-1-mediated depletion, we performed low-input RNA-seq using three independent biological replicates per group, with approximately 400 8-cell-stage embryos pooled for each library (approximately 1200 embryos per group; six libraries and approximately 2400 embryos in total). Using the nominal screening criteria of *p* ≤ 0.05 and |log2 fold change| ≥ 1, we identified 1212 candidate differentially expressed genes, including 285 upregulated and 927 downregulated genes (Figure 4A). These genes were subsequently used for exploratory pathway analysis and candidate-gene selection. We subsequently performed pathway enrichment analysis. KEGG pathway analysis of DEGs revealed that *LincSAKP* depletion inhibited the expression of transcription factors associated with ZGA (Figure 4B).

To validate the expression changes identified by pathway analysis, we collected 150 8-cell stage embryos injected with *LincSAKP* ASO-1 or NC ASO and performed RT–qPCR analysis. Based on the KEGG pathway analysis, we selected 10 representative candidate genes from the differentially expressed transcription factors for qPCR validation The expression levels of key genes involved in the identified signaling pathways, including *EP300*, *JUND*, *MEF2D*, *TCF15*, *THRA*, *TP73*, *YBX1*, *ZFP57*, *ZNF296*, and *ZNF746*, were examined. Among these genes, *EP300* expression was reduced by approximately two-fold in *LincSAKP*-depleted embryos compared with control embryos (Figure 4C), suggesting that *EP300* may play an important role during early porcine embryonic development. To determine whether the reduction in *EP300* transcript abundance following *LincSAKP* depletion was accompanied by a corresponding change at the protein level, we examined EP300 by immunofluorescence in 8-cell-stage embryos. Nuclear EP300 fluorescence was significantly reduced in *LincSAKP* ASO-1-treated embryos compared with NC ASO controls after accounting for experimental batch (Figure S5A,B). These results indicate that *LincSAKP* depletion is associated with reduced EP300 abundance at both the RNA and protein levels. Given that *EP300* showed a marked reduction in expression among the candidate transcriptional regulators identified by RNA-seq, served as a hub node in KEGG-enriched pathways, and is known as a key histone acetyltransferase and transcriptional coactivator, we therefore focused our subsequent functional analyses on *EP300*.

To investigate the function of *EP300* during early porcine embryonic development, *EP300* expression was knocked down using ASOs, and embryonic developmental competence was evaluated. ASOs were microinjected into embryos at the pronuclear stage, and blastocyst formation rates were assessed at 168 h after PA to compare embryonic development between the *EP300* ASO and NC ASO groups (Figure 2A). Four ASOs targeting *EP300* (*EP300* ASO-1, ASO-2, ASO-3, and ASO-4) were designed. Embryos were collected at the 8-cell stage after injection, and depletion efficiency was examined by RT–qPCR. Compared with the NC ASO group, *EP300* ASO-1, ASO-2, and ASO-3 achieved depletion efficiencies of approximately 80% (Figure 4D). Correspondingly, embryos exhibited developmental arrest, accompanied by a significant reduction in blastocyst formation at 168 h (Figure 4E). Further analysis of embryonic development demonstrated that depletion of *EP300* by ASO-1, ASO-2, or ASO-3 significantly reduced blastocyst formation rates in porcine embryos (Figure 4F and Figure S5C, Table 2 and Table S3). To exclude potential effects caused by microinjection, cleavage rates were assessed at 48 h after PA. The results showed that *EP300* ASO-1 to ASO-4 and NC ASO did not affect embryonic cleavage rates (Figure 4G and Figure S5C, Table 2 and Table S3). We further analyzed developmental arrest rates at different stages. Embryos injected with *EP300* ASO-1, ASO-2, or ASO-3 exhibited increased developmental arrest during the transition from the 4-cell to the 8-cell stage. Approximately 60% of embryos were arrested during the 4-cell to 8-cell transition, and nearly 40% were arrested at the 8-cell stage (Figure 4H and Figure S5C, Table 2 and Table S3). These results demonstrate that *EP300* plays an important regulatory role in ZGA during early porcine embryonic development.

### 3.5. EP300 Functions Downstream of LincSAKP in Regulating ZGA Progression

**Figure 5:**
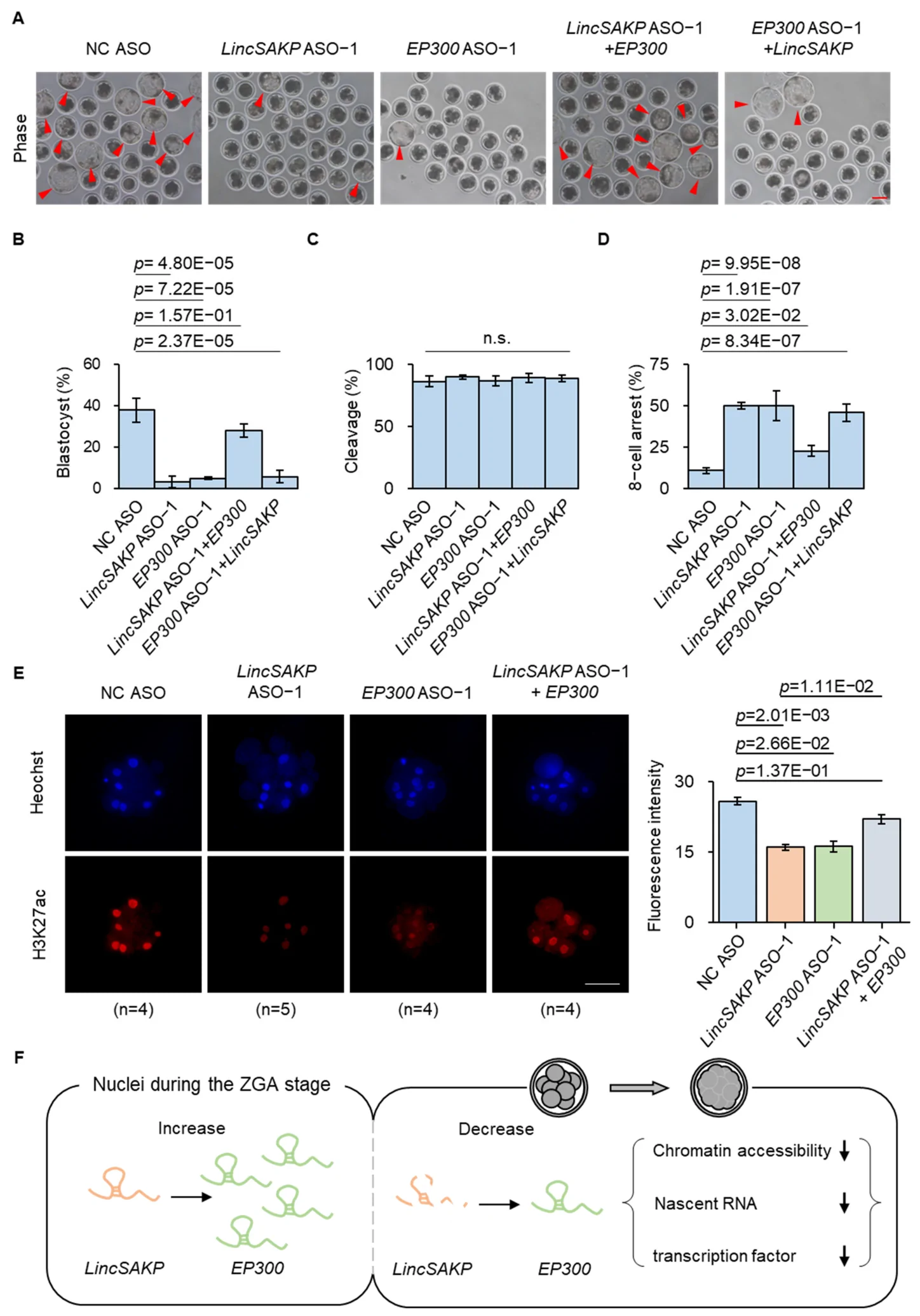
*EP300* is a key downstream regulatory factor of *LincSAKP*. (**A**) Overexpression of *EP300*, a key downstream factor of *LincSAKP*, rescues the developmental arrest caused by *LincSAKP* depletion. Photographs were taken at pPA 168 h (blastocyst stage). Scale bar, 100 μm. At least three experimental replicates were performed for each RNAi injection. (**B**) Analysis of blastocyst development: overexpression of *EP300* rescues the blastocyst developmental arrest induced by *LincSAKP* depletion. At least three experimental replicates were performed for each RNAi injection. (**C**) Analysis of cleavage development: embryos were injected with *LincSAKP* ASO-1, *EP300* ASO-1, *LincSAKP* ASO-1 + *EP300*, or *EP300* ASO-1 + *LincSAKP* at 6 h pPA, and the number of cleaved embryos was counted at 48 h of development to calculate the cleavage rate. There was no significant difference in the cleavage rate among the different treatment groups. At least three experimental replicates were performed for each RNAi injection. (**D**) Analysis of 8-cell arrest: overexpression of *EP300* rescues the significant increase in the 8-cell arrest rate caused by *LincSAKP* depletion. At least three experimental replicates were performed for each RNAi injection. (**E**) H3K27ac levels in 8-cell-stage embryos following *LincSAKP* depletion, *EP300* depletion, and *EP300* overexpression under *LincSAKP*-depleted conditions were assessed by immunofluorescence. Fluorescence measurements from all measurable nuclei within each embryo were averaged to obtain one embryo-level value, and each embryo was treated as one independent statistical unit. The NC ASO, *LincSAKP* ASO-1, *EP300* ASO-1, and *LincSAKP* ASO-1 + *EP300* groups contained 4, 5, 4, and 4 embryos, respectively, distributed across three independent biological batches as 2/1/1, 2/1/2, 1/1/2, and 1/1/2. Data are shown as mean ± SEM. Scale bar, 100 μm. (**F**) Proposed working model for the *LincSAKP*–*EP300* regulatory relationship during porcine ZGA. The model proposes that *LincSAKP* contributes to maintenance of *EP300* expression and H3K27ac, thereby supporting chromatin accessibility, nascent transcription, and ZGA-associated gene expression.

To investigate the regulatory relationship between *LincSAKP* and *EP300*, we constructed *LincSAKP* overexpression and *EP300* overexpression vectors and co-injected them with the corresponding *LincSAKP* or *EP300* ASOs into 1-cell stage embryos. Embryonic development was assessed at 168 h after PA. Compared with the positive control NC ASO group and the negative control groups injected with *LincSAKP* ASO-1 or *EP300* ASO-1 alone, co-injection of *LincSAKP* ASO-1 and the *EP300* overexpression vector rescued embryonic development, with some embryos successfully reaching the blastocyst stage. In contrast, co-injection of *EP300* ASO-1 and the *LincSAKP* overexpression vector failed to rescue embryonic development, and blastocyst formation remained impaired (Figure 5A).

Consistently, analysis of blastocyst formation rates showed that *EP300* overexpression significantly rescued the developmental defects caused by *LincSAKP* depletion (Figure 5B and Figure S6A, Table 3 and Table S4). Similarly, assessment of cleavage rates demonstrated that injection of different combinations did not affect embryonic cleavage, indicating that the observed developmental defects were not caused by microinjection procedures (Figure 5C and Figure S6A, Table 3 and Table S4). Analysis of developmental arrest at different embryonic stages further revealed that the 8-cell stage arrest induced by *LincSAKP* depletion was alleviated by *EP300* overexpression, resulting in partial recovery of embryonic development (Figure 5D and Figure S6A, Table 3 and Table S4). To further confirm *LincSAKP* overexpression in the reciprocal rescue experiment, we examined *LincSAKP* abundance and distribution in 8-cell-stage embryos injected with *EP300* ASO-1 alone or co-injected with *EP300* ASO-1 + *LincSAKP*. RNA-FISH showed a stronger *LincSAKP* signal in the co-injection group, with predominant nuclear enrichment (Figure S6B). Consistently, qPCR analysis showed a significant increase in total *LincSAKP* expression in embryos co-injected with *EP300* ASO-1 + *LincSAKP* (Figure S6C). Because the FISH probes recognize sequences shared by endogenous and injected *LincSAKP*, these assays detect total *LincSAKP* and do not distinguish the two transcript sources. Thus, the increased FISH signal and qPCR abundance confirm effective *LincSAKP* overexpression in the rescue group, while the observed nuclear enrichment reflects the distribution of total *LincSAKP*. Together with the reciprocal rescue results, these findings support a model in which *EP300* acts downstream of *LincSAKP* during ZGA progression.

To further examine whether *EP300* mediates the effect of *LincSAKP* on histone acetylation, we quantified H3K27ac levels in 8-cell-stage embryos. After accounting for experimental batch, H3K27ac fluorescence was significantly reduced following either *LincSAKP* ASO-1 or *EP300* ASO-1 treatment compared with NC ASO controls. Importantly, *EP300* overexpression in *LincSAKP*-depleted embryos significantly increased H3K27ac fluorescence compared with *LincSAKP* ASO-1 treatment alone, restoring the signal toward the level observed in NC ASO embryos (Figure 5E). These findings provide additional molecular rescue evidence supporting *EP300* as a downstream mediator of the H3K27ac changes associated with *LincSAKP* depletion.

Collectively, these findings support a model in which *LincSAKP* contributes to porcine ZGA, at least in part, through *EP300*-associated regulation. *LincSAKP* ASO-1-mediated depletion was accompanied by reduced *EP300* expression and H3K27ac, decreased chromatin accessibility and nascent transcription, and altered expression of ZGA-associated transcription factors. Together with the partial developmental rescue by *EP300* overexpression, these findings support *EP300* as a downstream mediator and form the basis of the proposed *LincSAKP*–*EP300* regulatory model during ZGA (Figure 5F). This model provides a framework for understanding the potential regulatory role of *LincSAKP* during porcine early PA embryonic development.

## 4. Discussion

In this study, we identified a novel SINE-associated lncRNA, named *LincSAKP*, and demonstrated its important role in zygotic genome activation in porcine parthenogenetically activated embryos. Our results showed that *LincSAKP* is a nuclear-localized non-coding RNA. *LincSAKP* expression reaches its peak at the 8-cell stage in porcine embryos, and depletion of *LincSAKP* impairs ZGA progression. Further functional analyses revealed that *EP300* functions downstream of *LincSAKP* in the regulatory pathway, as evidenced by the similar developmental phenotypes upon *EP300* depletion and the partial rescue of *LincSAKP* knockdown defects by *EP300* overexpression. Depletion of *LincSAKP* or *EP300* affects histone H3K27ac modification. Collectively, this study identifies *LincSAKP* as a functional SINE-containing lncRNA involved in porcine ZGA and provides a basis for further investigation of SINE-containing lncRNAs during early PA embryonic development. However, this study has certain limitations. Although PA embryos can effectively recapitulate ZGA and other early events, they contain only the maternal genome and lack paternal alleles, potentially leading to imprinting abnormalities that may restrict later development. Thus, the conclusion that *LincSAKP* regulates *EP300* should be extrapolated to normal development with caution, and further validation in fertilized embryos is needed. In addition, although the ASO-resistant rescue supports the specificity of the developmental phenotype, the downstream molecular phenotypes were not independently validated by rescue or a second effective ASO, and their on-target specificity therefore requires further confirmation.

During ZGA, transposable elements (TEs) often function as enhancers or promoters to drive the transcription of themselves and their fused sequences. Alternatively, TEs can generate functional non-coding RNAs, including TE-associated lncRNAs. These RNAs, such as *MLT2A1* chimeric transcripts [31,35] and *LincGET* [13,14,15], act as key regulatory molecules rather than simple transcriptional by-products. Through the establishment of multilayered regulatory networks involving transcriptional activation, amplification, and target localization, these TE-derived RNAs collectively ensure the robust progression of ZGA, a critical developmental event [43,44,45]. Investigation of their regulatory mechanisms contributes to a better understanding of the complexity underlying ZGA regulation. In this study, we identified LincSAKP as a SINE-containing lncRNA that is dynamically expressed during porcine ZGA and participates in the regulation of ZGA-associated genes. These findings identify *LincSAKP* as a porcine SINE-containing lncRNA associated with ZGA regulation and provide a basis for investigating whether its SINE-derived sequences contribute to this function.

Previous studies have shown that some TE-associated lncRNAs can function as transcriptional regulators of ZGA-associated genes. For example, the endogenous retrovirus MuERV-L plays a critical driving role in the activation of lncRNAs associated with minor ZGA, and these lncRNAs are essential regulators during ZGA [46]. *LincGET* is indispensable for mouse ZGA and has been considered a major regulator of ZGA during the 2-cell stage in mice [13]. In pigs, *SAWPA* regulates JNK transcription via its SINE sequence in trans, thereby modulating ZGA progression [32]. In addition, TE-associated lncRNAs can serve as central components of “chimeric RNA” networks, driving global transcriptional activation and functioning as cis-regulatory elements to remodel chromatin and initiate transcription. The human ERV subfamily *MLT2A1* is not transcribed as an intact ERV element but instead forms diverse chimeric RNAs through fusion with downstream sequences, such as *LINE1* and Alu elements. The conserved 5′ region of these transcripts binds the nuclear protein HNRNPU and recruits RNA polymerase II, providing transcriptional activation capacity, whereas the diverse 3′ sequences mediate recognition and binding to a broad range of genomic loci. These diverse chimeric RNAs cooperatively activate ZGA regulatory networks and promote ZGA progression [31]. In this study, we found that *LincSAKP*, a SINE-containing lncRNA, is functionally linked to *EP300* in the regulation of ZGA. EP300 is a histone acetyltransferase and transcriptional coactivator. The observation that both *LincSAKP* and *EP300* depletion reduce H3K27ac levels and chromatin accessibility suggests that *LincSAKP* and EP300 may act within a common pathway to influence the epigenetic landscape during ZGA. These findings suggest a regulatory link between *LincSAKP* and *EP300* during ZGA. However, the present study does not determine whether the MIR or Pre0 sequences contribute to this regulatory activity. Future studies employing targeted deletion or mutation of the MIR and Pre0 regions will be required to determine whether these SINE-derived sequences are functionally involved in *LincSAKP*-mediated regulation.

Transcription factors (TFs), as core molecular recognition components of gene regulatory networks, recognize specific nucleotide motifs within cis-regulatory elements through their DNA-binding domains and subsequently recruit epigenetic regulatory complexes and transcriptional effectors, such as RNA polymerase II (Pol II), to establish gene expression programs [47]. Therefore, TFs represent essential components of transcriptional regulation during development. ZGA is accompanied by the establishment of extensive autonomous transcription from the embryonic genome, during which numerous transcription factors are transcriptionally activated and participate in regulating ZGA progression. Dppa2/4 function as positive regulators to activate ZGA transcription, whereas Alppl2 may promote ZGA progression through folate metabolism [48]. The DUX family (mouse DUX/human DUX4) is expressed during ZGA and cooperatively activates core ZGA genes by recruiting chromatin remodeling complexes [49]. In mice, DUX depletion blocks the transition to the 2-cell-like state, and conditional knockout models have demonstrated that DUX deficiency results in developmental arrest during ZGA, silencing of core ZGA genes, and abnormal chromatin accessibility, indicating that DUX is a key regulator of mammalian ZGA [50]. In addition, *Zelda* in *Drosophila* [51] and NR5A2 in mice [23] have also been identified as important ZGA regulators. Among them, NR5A2 has been demonstrated to be essential for ZGA through siRNA-mediated depletion, protein degradation, and chemical inhibition approaches [24]. These findings collectively demonstrate the direct regulatory relationship between transcription factors and ZGA. In this study, we found that depletion of *LincSAKP* affected the expression of numerous transcription factors during ZGA, including *EP300* and *ZSCAN4*. *EP300* can be recruited to genomic regions by the key ZGA pioneer factor DUX, where it cooperatively activates downstream gene expression [52]. In mice, *ZSCAN4* is a marker gene specifically expressed during the 2-cell stage and participates in embryonic genome reprogramming by regulating the expression of ZGA-associated genes [53]. During early porcine embryonic development, *ZSCAN4* regulates ZGA progression, represses DNMT1 expression, and contributes to telomere length maintenance [54]. These findings provide new insights into the regulatory mechanisms underlying *LincSAKP*-mediated ZGA regulation. Although our results demonstrate that *LincSAKP* affects ZGA progression at least in part through modulation of *EP300* expression, this does not formally prove that *EP300* is the exclusive downstream mediator. Given that RNA-seq analysis revealed altered expression of multiple transcription factors and signaling pathway components upon *LincSAKP* depletion, the possibility of additional downstream effectors cannot be excluded. Moreover, overexpression of *EP300* could only compensate for a portion of the functional defects caused by *LincSAKP* depletion, and was unable to fully reverse all downstream perturbations. Future studies employing targeted perturbation of other candidate genes will be required to comprehensively delineate the downstream regulatory network of *LincSAKP*.

P300, encoded by the *EP300* gene, is a highly conserved histone acetyltransferase (HAT) in eukaryotic cells [55]. Through catalyzing the acetylation of histone and non-histone proteins, p300 participates in key biological processes, including cell growth, differentiation, development, and stress responses [56,57]. Acetylation modifications mediated by the p300/CBP complex establish an open chromatin structure, which provides a fundamental basis for ZGA initiation [58,59]. Studies have demonstrated that the catalytic activity of p300/CBP is indispensable during minor ZGA, and loss of its function results in mouse embryonic arrest at the 2-cell stage [55]. Meanwhile, p300/CBP cooperates with histone deacetylases (HDACs) to regulate the dynamic balance of H3K27 acetylation and deacetylation, thereby promoting the initiation of minor ZGA while preventing premature activation of developmental genes, ensuring proper progression of ZGA [60]. Acetylation modifications play critical roles in ZGA regulation, and their regulatory mechanisms have become a major focus in the field of early embryonic development. At the metabolic level, NAD^+^ activates the deacetylase SIRT1 to remove fertilization-derived H3K27ac, ensuring the timely silencing of minor ZGA genes. This mechanism directly links metabolic status with epigenetic regulation to maintain normal embryonic development [61]. In early porcine embryos, nuclear accumulation of pyruvate dehydrogenase E1 component subunit alpha 1 (*PDHA1*) promotes histone acetylation and is essential for ZGA. In addition, the mitochondrial enzyme *ACSS1* converts acetate into acetyl-CoA, which is utilized for energy production through the tricarboxylic acid (TCA) cycle and generation of metabolic intermediates. *ACSS1* also regulates ZGA through modulation of histone acetylation, further highlighting the central role of histone acetylation in ZGA regulation [62,63]. In this study, we found that *LincSAKP* regulates ZGA progression through modulation of *EP300* expression, and that *EP300* overexpression rescues the embryonic developmental arrest caused by *LincSAKP* depletion. These findings suggest that *LincSAKP* may regulate gene transcription during porcine ZGA through modulation of acetylation modifications. In addition, H3K27ac is a well-established marker of active enhancers and promoters, and its dynamic changes are closely associated with transcriptional activation [64]. Given that the relationship between H3K27ac and porcine ZGA remains insufficiently characterized, our findings provide a new entry point and theoretical basis for further elucidating the regulatory mechanisms of acetylation modifications during early porcine embryonic development.

Embryonic cells possess remarkable plasticity—the potential to generate various cell types upon differentiation—which is essential for initiating complete developmental programs [65]. During early embryogenesis, dynamic chromatin remodeling underlies the transition of cellular plasticity, with increased chromatin accessibility accompanying both ZGA and the first lineage segregation (ICM formation), while higher chromatin mobility marks a totipotent nuclear state [66]. Abundant retrotransposons in the genome facilitate chromatin opening. For example, ERV-related lincRNAs such as *LincGET* enhance chromatin accessibility through CARM1-mediated H3R26me2 deposition [14], and LINE1 expression has been reported to increase chromatin accessibility in mouse 2-cell embryos [67], suggesting that retrotransposons play important roles in chromatin regulation. Regarding the SINE-associated lncRNA *LincSAKP*, we found that its depletion reduced chromatin accessibility, accompanied by decreased *EP300* expression, implying that *LincSAKP* may modulate histone acetylation to influence chromatin openness, although further experiments—such as SINE deletion or mutation analyses—are required to determine whether the SINE sequences directly contribute to this regulatory function. Nevertheless, the DNase I-TUNEL assay used in this study has limitations in resolution compared with genome-wide approaches such as ATAC-seq. Thus, ATAC-seq will be a focus of future investigations to provide more comprehensive and high-resolution chromatin accessibility profiling.

The regulation of target genes by lncRNAs is essential for elucidating the mechanisms underlying ZGA and identifying potential therapeutic targets. Current studies have revealed that lncRNAs regulate target genes mainly through two modes of action: cis regulation and trans regulation, and they can function at the epigenetic, transcriptional, and post-transcriptional levels [68,69]. In cis regulation, lncRNAs regulate genes located near their own genomic loci, often by recruiting chromatin-modifying complexes, such as PRC2, to alter local histone modification states, or by functioning as enhancer RNAs to directly activate the transcription of neighboring genes [70,71,72,73]. In this study, depletion of *LincSAKP* did not affect the expression of its neighboring genes *KTN1* and *PELI2*, suggesting that *LincSAKP* may regulate ZGA through a trans-acting mechanism. Through trans regulation, lncRNAs can function independently of their transcriptional loci. They can act as molecular decoys to sequester transcription factors, serve as molecular scaffolds to assemble ribonucleoprotein complexes, function as miRNA sponges to regulate mRNA stability, or influence mRNA splicing, translation, or degradation through base pairing with target mRNAs [74,75,76,77]. In this study, we identified *EP300* as a key downstream target of *LincSAKP* and preliminarily demonstrated that *LincSAKP* regulates a critical ZGA-associated target gene through a trans-acting mechanism. Furthermore, *LincSAKP* contains a 19-bp complementary sequence with *EP300*. However, the precise molecular mechanism by which *LincSAKP* regulates *EP300* remains to be further investigated. It remains unclear whether *LincSAKP* regulates *EP300* transcription or translation through direct base pairing with *EP300* transcripts. In future studies, RNA pull-down assays and co-immunoprecipitation (Co-IP) experiments will be performed to further explore and validate the regulatory mechanism of *LincSAKP*. In addition, luciferase reporter assays will be used to determine whether the SINE element contributes to the regulatory function of *LincSAKP* on its target gene *EP300*. These studies will further elucidate the mechanisms by which SINE-associated lncRNAs participate in zygotic genome activation during porcine embryonic development.

In summary, this study identifies *LincSAKP* as a SINE-associated lncRNA that participates in zygotic genome activation in porcine parthenogenetically activated embryos. Our data support *EP300* as a downstream mediator of *LincSAKP* in this regulatory process, supported by epistatic rescue experiments and shared effects on H3K27ac levels. These findings expand our understanding of the regulatory role of *LincSAKP* in transcriptional reprogramming during porcine preimplantation embryonic development and provide an experimental foundation for further elucidating the molecular mechanisms by which *LincSAKP* regulates early porcine embryonic development.

## Figures and Tables

**Table 1:** Statistical analysis of embryonic development following LincSAKP depletion.

Group	Experiments (n)	Embryos (n)	2C Arrest (%)	MO Arrest (%)	8C Arrest (%)	4−8C Arrest (%)	BL (%)
NC ASO	3	78	5.6 ± 3.0	11.2 ± 4.1	15.5 ± 0.9	26.9 ± 3.4	39.5 ± 3.1
*LincSAKP* ASO-1	3	87	11.5 ± 4.6	13.9 ± 4.2	38.9 ± 5.7	56.1 ± 7.4	2.3 ± 1.2
*LincSAKP* ASO-2	3	77	3.8 ± 2.1	11.5 ± 1.7	41.8 ± 3.5	60.2 ± 5.2	5.3 ± 1.5
*LincSAKP* ASO-3	3	101	6.9 ± 0.5	14.2 ± 2.9	16.0 ± 1.5	32.8 ± 1.9	32.6 ± 1.9
*LincSAKP* ASO-4	3	97	9.4 ± 2.1	13.3 ± 2.2	39.2 ± 2.3	55.6 ± 3.7	7.0 ± 2.4

2C, 2-cell stage; 4C, 4-cell stage; 4–8C, 4- to 8-cell stage; MO, morula stage; BL, blastocyst stage. Data are shown as mean ± SEM from three independent biological replicates (independent embryo-culture batches). Developmental outcomes were analyzed using binomial GLMMs with a logit link, with Treatment as a fixed effect and Batch as a random intercept. Pairwise *p* values and 95% confidence intervals are provided in Figure S2 and Table S2, respectively. Total embryo numbers are indicated in the “Embryos (*n*)” column.

**Table 2:** Statistical analysis of embryonic development following EP300 depletion.

Group	Experiments (n)	Embryos (n)	2C Arrest (%)	MO Arrest (%)	8C Arrest (%)	4−8C Arrest (%)	BL (%)
NC ASO	3	87	5.5 ± 1.7	11.8 ± 2.1	19.7 ± 1.1	36.1 ± 3.9	39.9 ± 2.4
*EP300* ASO-1	3	77	9.3 ± 3.0	13.6 ± 2.8	46.4 ± 1.8	63.2 ± 3.2	7.2 ± 2.8
*EP300* ASO-2	3	87	9.9 ± 3.0	14.2 ± 3.1	43.8 ± 1.7	59.0 ± 3.2	2.4 ± 1.2
*EP300* ASO-3	3	76	8.9 ± 3.0	8.4 ± 3.1	47.4 ± 7.2	67.1 ± 7.7	6.8 ± 1.7
*EP300* ASO-4	3	82	4.9 ± 0.2	9.9 ± 2.9	15.6 ± 2.7	34.4 ± 3.9	37.4 ± 9.7

2C, 2 cell stage; 4C, 4 cell stage; 4 8C, 4- to 8-cell stage; MO, morula stage; BL, blastocyst stage. Data are shown as mean ± SEM from three independent biological replicates (independent embryo-culture batches). Developmental outcomes were analyzed using binomial GLMMs with a logit link, with Treatment as a fixed effect and Batch as a random intercept. Pairwise *p* values and 95% confidence intervals are provided in Figure S5 and Table S3, respectively. Total embryo numbers are indicated in the “Embryos (*n*)” column.

**Table 3:** Statistical analysis of embryonic development following dual depletion of LincSAKP and EP300 and rescue experiments.

Group	Experiments (n)	Embryos (n)	2C Arrest (%)	MO Arrest (%)	8C Arrest (%)	4−8C Arrest (%)	BL (%)
NC ASO	3	103	9.8 ± 3.6	13.6 ± 4.3	10.7 ± 0.9	25.2 ± 2.4	37.9 ± 3.4
*LincSAKP* ASO-1	3	70	12.0 ± 3.7	7.1 ± 2.5	50.0 ± 1.1	67.6 ± 2.4	3.2 ± 1.7
*EP300* ASO-1	3	62	4.9 ± 0.3	9.5 ± 2.4	50.0 ± 5.3	67.6 ± 2.2	4.9 ± 0.3
*LincSAKP* ASO-1 + *EP300*	3	79	6.4 ± 2.6	16.6 ± 0.8	22.6 ± 1.9	38.3 ± 5.1	28.2 ± 1.8
*EP300* ASO-1 + *LincSAKP*	3	72	10.8 ± 3.4	8.4 ± 0.4	46.0 ± 3.1	63.9 ± 0.1	5.7 ± 1.7

2C, 2-cell stage; 4C, 4-cell stage; 4–8C, 4- to 8-cell stage; MO, morula stage; BL, blastocyst stage. Data are shown as mean ± SEM from three independent biological replicates (independent embryo-culture batches). Developmental outcomes were analyzed using binomial GLMMs with a logit link, with Treatment as a fixed effect and Batch as a random intercept. Pairwise *p* values and 95% confidence intervals are provided in Figure S6 and Table S4, respectively. Total embryo numbers are indicated in the “Embryos (*n*)” column.

## Data Availability

The raw RNA-seq data generated in this study have been deposited in the Genome Sequence Archive (GSA) at the National Genomics Data Center, China National Center for Bioinformation (CNCB), under BioProject accession number PRJCA073382. The other original contributions presented in this study are included in the article/Supplementary Materials. Further inquiries can be directed to the corresponding authors.

## Supplementary Materials

The following supporting information can be downloaded at https://www.mdpi.com/article/10.3390/cells15181707/s1, Figure S1: Primer sequence information for *LincSAKP* and analysis of its protein-coding potential; Figure S2: Pairwise comparison *p* values for embryonic developmental outcomes following *LincSAKP* ASO 1–4 microinjection and validation using *LincSAKP* ASO-1.; Figure S3: The negative control groups for the DNase I-TUNEL assay and EU labeling experiments; Figure S4: Depletion of *LincSAKP* does not affect the expression of its neighboring genes. Figure S5: validation of reduced EP300 protein levels following *LincSAKP* depletion and Pairwise comparison *p* values for embryonic developmental outcomes following *EP300* ASO 1–4 microinjection.; Figure S6: Pairwise comparison *p* values for embryonic developmental outcomes in the rescue experiments and validation of *LincSAKP* overexpression; Table S1: Sequence of primers; Table S2: Binomial generalized linear mixed-effects model analysis of embryonic developmental outcomes following *LincSAKP* depletion; Table S3: Binomial generalized linear mixed-effects model analysis of embryonic developmental outcomes following *EP300* depletion; Table S4: Binomial generalized linear mixed-effects model analysis of embryonic developmental outcomes in the rescue experiments.
