# Supplementary material for: A Novel SINE Transposable Element-Associated lncRNA Regulates Zygotic Genome Activation in Porcine Parthenogenetically Activated Embryos
Source: Cells. 2026 Sep 19;15(18):1707. doi: 10.3390/cells15181707 (PMC13605572; doi:10.3390/cells15181707)
Supplement: Supplementary file 1 [file cells-15-01707-s001.zip › cells-4498686-supplementary.pdf]

cells

Supporting Information

A Novel SINE Transposable Element-Associated lncRNA Regulates Zygotic Genome Activation in Porcine Parthenogenetically Activated Embryos

Tianyao He[1, #], Shanlong Zhang[1, #], Shichao Zhang[1], Dongsong Liu[1], Han Li[1], Jun Song[1], Zhonghua Liu[1*], Jun-Xue Jin[1*], Jing-Tao Sun[1*]

Figure S1–S6

Table S1–S4

 https://doi.org/10.3390/cells15181707

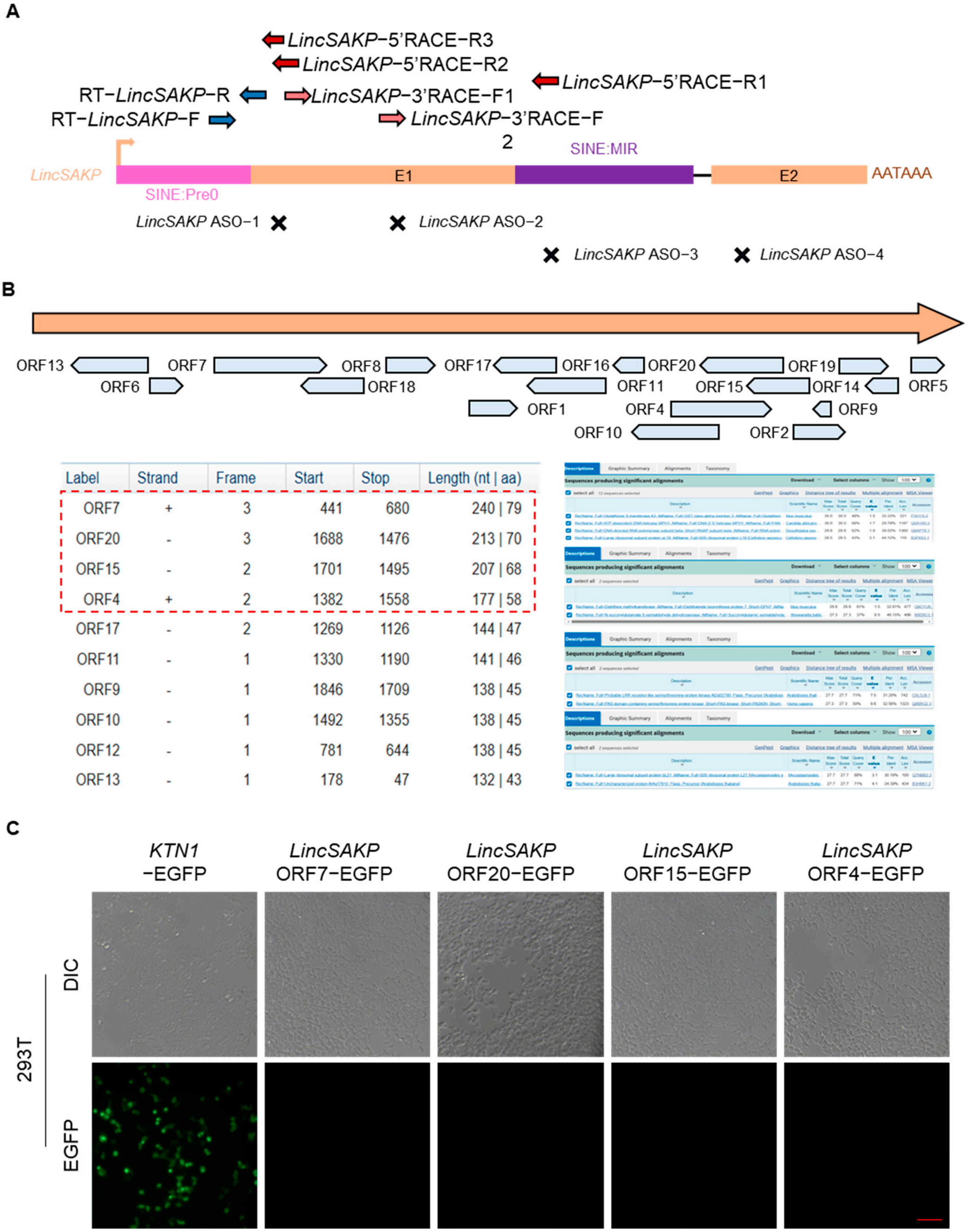

Label	Strand	Frame	Start	Stop	Length (nt | aa)
ORF7	+	3	441	680	240 | 79
ORF20	-	3	1688	1476	213 | 70
ORF15	-	2	1701	1495	207 | 68
ORF4	+	2	1382	1558	177 | 58
ORF17	-	2	1269	1126	144 | 47
ORF11	-	1	1330	1190	141 | 46
ORF9	-	1	1846	1709	138 | 45
ORF10	-	1	1492	1355	138 | 45
ORF12	-	1	781	644	138 | 45
ORF13	-	1	178	47	132 | 43

Figure S1. Primer sequence information for *LincSAKP* and analysis of its protein-coding potential. **A)** Positions of qPCR primers, RACE-related primers, and interference fragments on *LincSAKP*. *LincSAKP* contains SINE: MIR and Pre0 repetitive sequences. **B)** Prediction analysis of the coding potential of *LincSAKP*. The ORF represents the predicted region with possible protein-coding capacity. **C)** Detection of *LincSAKP* coding potential. EGFP without the ATG start codon was fused with ORF7, ORF20, ORF15, and ORF4 sequences,

and the fusion constructs were expressed in 293T cells followed by fluorescence intensity detection. Scale bar, 50 μm. Three experimental replicates were used.

https://doi.org/10.3390/cells15181707

A

BL (%)					
	NC ASO	*LincSAKP* ASO−1	*LincSAKP* ASO−2	*LincSAKP* ASO−3	*LincSAKP* ASO−4
NC ASO	1.00E+00	9.27E−06	9.99E−06	3.28E−01	2.69E−06
LincSAKP ASO−1		1.00E+00	3.37E−01	4.99E−05	1.43E−01
LincSAKP ASO−2			1.00E+00	8.65E−05	5.88E−01
LincSAKP ASO−3				1.00E+00	4.05E−05
LincSAKP ASO−4					1.00E+00

8C arrest (%)					
	NC ASO	*LincSAKP* ASO−1	*LincSAKP* ASO−2	*LincSAKP* ASO−3	*LincSAKP* ASO−4
NC ASO	1.00E+00	9.98E−04	4.68E−04	9.33E−01	7.81E−04
LincSAKP ASO−1		1.00E+00	7.47E−01	4.61E−04	9.90E−01
LincSAKP ASO−2			1.00E+00	2.00E−04	7.50E−01
LincSAKP ASO−3				1.00E+00	3.33E−04
LincSAKP ASO−4					1.00E+00

4C arrest (%)					
	NC ASO	*LincSAKP* ASO−1	*LincSAKP* ASO−2	*LincSAKP* ASO−3	*LincSAKP* ASO−4
NC ASO	1.00E+00	3.02E−01	2.48E−01	3.21E−01	3.54E−01
LincSAKP ASO−1		1.00E+00	8.75E−01	9.41E−01	8.93E−01
LincSAKP ASO−2			1.00E+00	8.14E−01	7.70E−01
LincSAKP ASO−3				1.00E+00	9.49E−01
LincSAKP ASO−4					1.00E+00

4−8C arrest (%)					
	NC ASO	*LincSAKP* ASO−1	*LincSAKP* ASO−2	*LincSAKP* ASO−3	*LincSAKP* ASO−4
NC ASO	1.00E+00	1.80E−04	5.44E−05	4.06E−01	1.77E−04
LincSAKP ASO−1		1.00E+00	6.58E−01	1.25E−03	9.29E−01
LincSAKP ASO−2			1.00E+00	3.82E−04	5.90E−01
LincSAKP ASO−3				1.00E+00	1.25E−03
LincSAKP ASO−4					1.00E+00

Cleavage (%)					
	NC ASO	*LincSAKP* ASO−1	*LincSAKP* ASO−2	*LincSAKP* ASO−3	*LincSAKP* ASO−4
NC ASO	1.00E+00	9.21E−01	6.49E−01	6.03E−01	6.85E−01
LincSAKP ASO−1		1.00E+00	5.71E−01	6.69E−01	7.55E−01
LincSAKP ASO−2			1.00E+00	3.17E−01	3.76E−01
LincSAKP ASO−3				1.00E+00	9.08E−01
LincSAKP ASO−4					1.00E+00

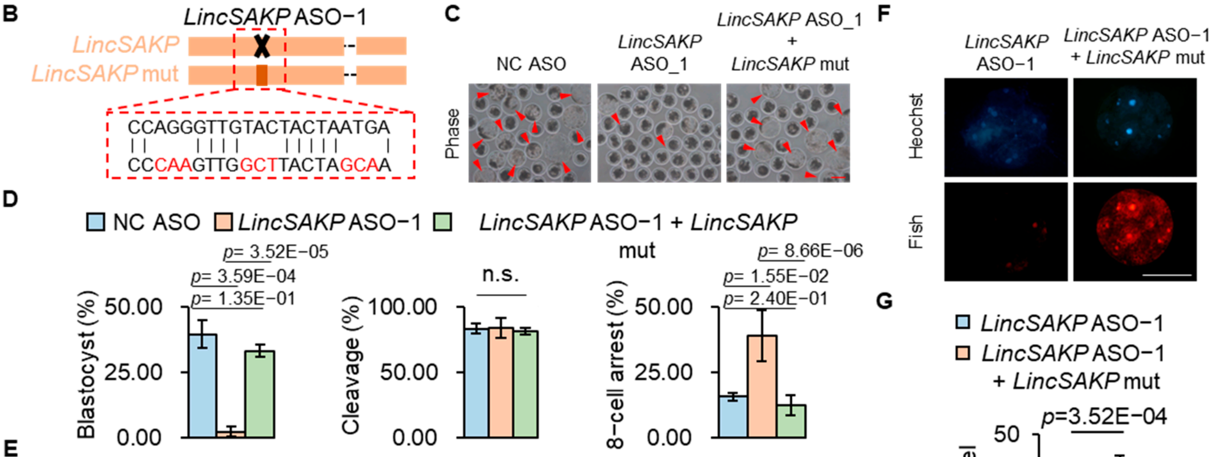

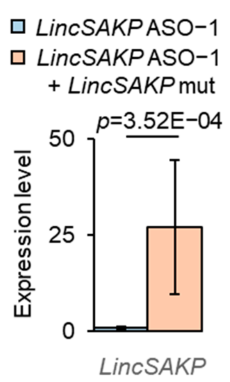

E

Endpoint	Direct rescue contrast	OR	95% CI	Exact P
BL	ASO−1 + LincSAKP−mut vs ASO−1	0.05	0.01−0.20	3.52E−5
8C arrest	ASO−1 + LincSAKP−mut vs ASO−1	4.65	2.36−9.17	8.66E−6
4−8C arrest	ASO−1 + LincSAKP−mut vs ASO−1	3.08	1.75−5.41	9.85E−5
Cleavage	ASO−1 + LincSAKP−mut vs ASO−1	1.22	0.59−2.50	5.90E−1

Figure S2. Pairwise comparison *P* values for embryonic developmental outcomes following *LincSAKP* ASO 1–4 microinjection and validation using *LincSAKP* ASO-1. **A)** Pairwise comparison *P* values for blastocyst formation, 8-cell arrest, 4-cell arrest, 4- to 8-cell arrest, and cleavage following *LincSAKP* ASO 1–4

https://doi.org/10.3390/cells15181707

microinjection. P values were obtained from pairwise contrasts based on binomial generalized linear mixed-effects models (GLMMs), with Treatment as a fixed effect and Batch as a random intercept. P < 0.05 was considered statistically significant. **B)** Schematic representation of the ASO-1-resistant *LincSAKP* mutant (*LincSAKP* mut). The nucleotide substitutions were introduced within the ASO-1-binding region. The wild-type and mutant sequences are shown, with substituted nucleotides highlighted in red. **C)** Overexpression of *LincSAKP* mut rescues the embryonic developmental arrest caused by *LincSAKP* depletion. Photographs were taken at 168 h post-parthenogenetic activation at the blastocyst stage. Scale bar, 100 µm. At least three independent biological replicates were performed for each injection group. **D)** Overexpression of *LincSAKP* mut rescues the reduced blastocyst rate and the increased 8-cell arrest rate caused by *LincSAKP* depletion, without affecting the cleavage rate. At least three independent biological replicates were performed for each injection group. **E)** Direct statistical contrasts between LincSAKP ASO-1 and *LincSAKP* ASO-1 + *LincSAKP* mut. Odds ratios, 95% confidence intervals, and exact P values were obtained using binomial GLMMs with Treatment as a fixed effect and Batch as a random intercept. *LincSAKP* ASO-1 + *LincSAKP* mut was used as the reference group. **F)** RNA-FISH detection of *LincSAKP* in 8-cell-stage embryos injected with *LincSAKP* ASO-1 alone or co-injected with *LincSAKP* ASO-1 + *LincSAKP* mut. Nuclei were counterstained with Hoechst. Scale bar, 100 µm. Three independent biological replicates were performed. **G)** RT-qPCR analysis of *LincSAKP* RNA abundance in 8-cell-stage embryos injected with *LincSAKP* ASO-1 alone or co-injected with *LincSAKP* ASO-1 + *LincSAKP* mut. Error bars represent S.E.M. Approximately 50 embryos per group were used for each qPCR analysis, with three independent biological replicates. 18S rRNA served as the internal reference gene.

https://doi.org/10.3390/cells15181707

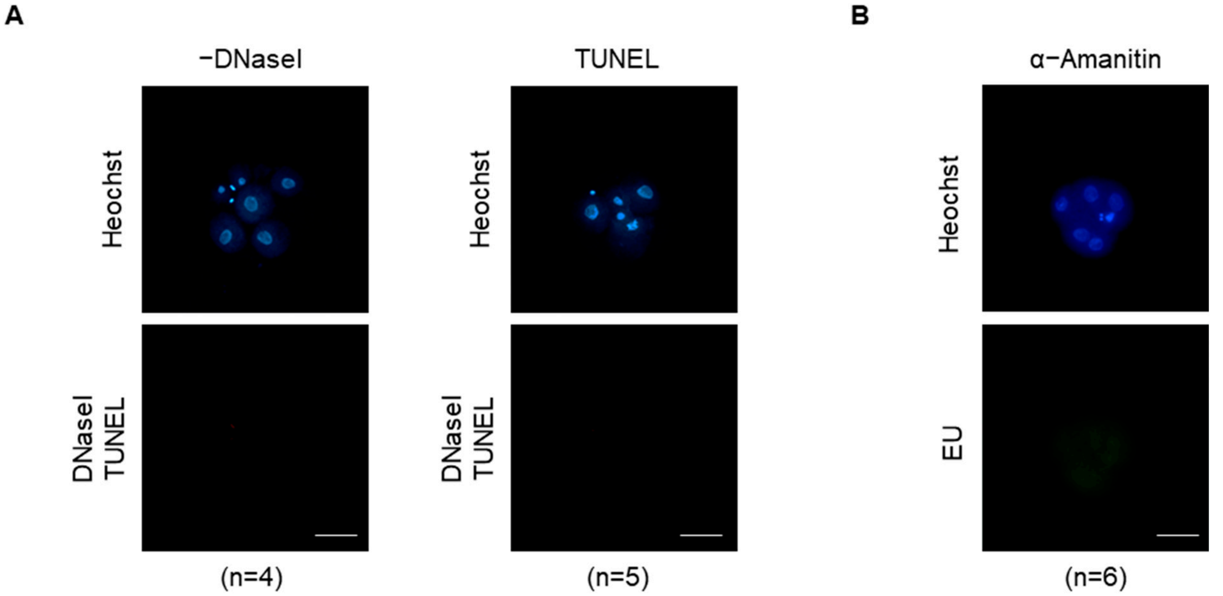

Figure S3. The negative control groups for the DNase I-TUNEL assay and EU labeling experiments. **A)** Fluorescence detection of the no-DNase control and TUNEL baseline control in 8-cell embryos by DNase I-TUNEL assay. Scale bar, 100 μm. **B)** Fluorescence detection of the transcription inhibition control in 8-cell embryos treated with α-amanitin during EU labeling. Scale bar, 100 μm.

https://doi.org/10.3390/cells15181707

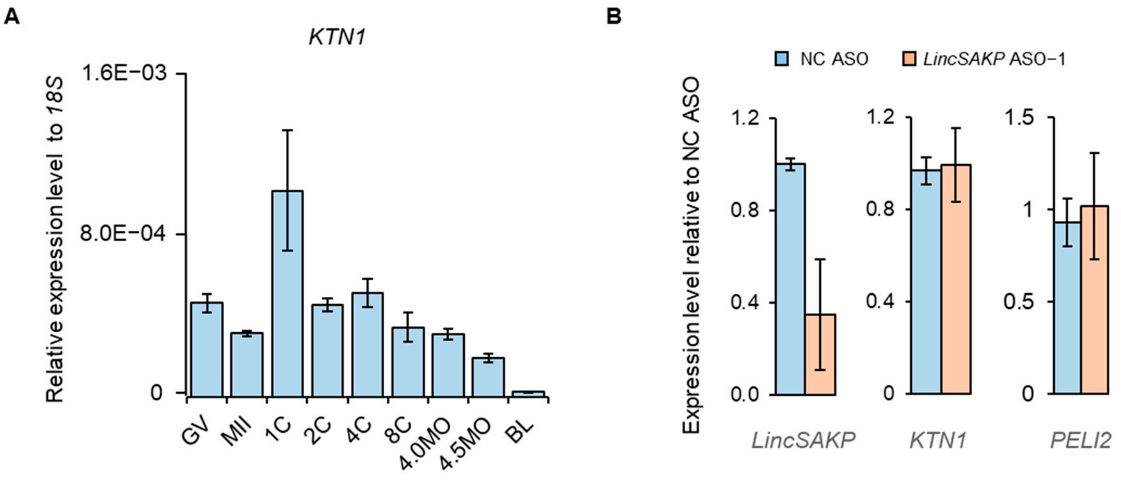

Figure S4. Depletion of *LincSAKP* does not affect the expression of its neighboring genes. **A)** RNA expression pattern of *KTN1* during porcine early embryonic development from oocyte to blastocyst, determined by qPCR. GV, germinal vesicle oocyte; 1C, 1-cell stage; 2C, 2-cell stage; 4C, 4-cell stage; 8C, 8-cell stage; 4D, 4-day morula; 4.5D, 4.5-day morula; BL, 7-day blastocyst. Error bars represent S.E.M. Approximately 50 embryos of each stage were used, and three experimental replicates were performed. **B)** Expression levels of *LincSAKP*, *KTN1*, and *PELI2* in 8-cell-stage embryos. After injection of *LincSAKP* ASO-1, the RNA levels of *LincSAKP*, *KTN1*, and *PELI2* were examined by qPCR. Error bars represent S.E.M. About 50 embryos of each stage were used, and three experimental replicates were performed. 18S served as an internal reference gene.

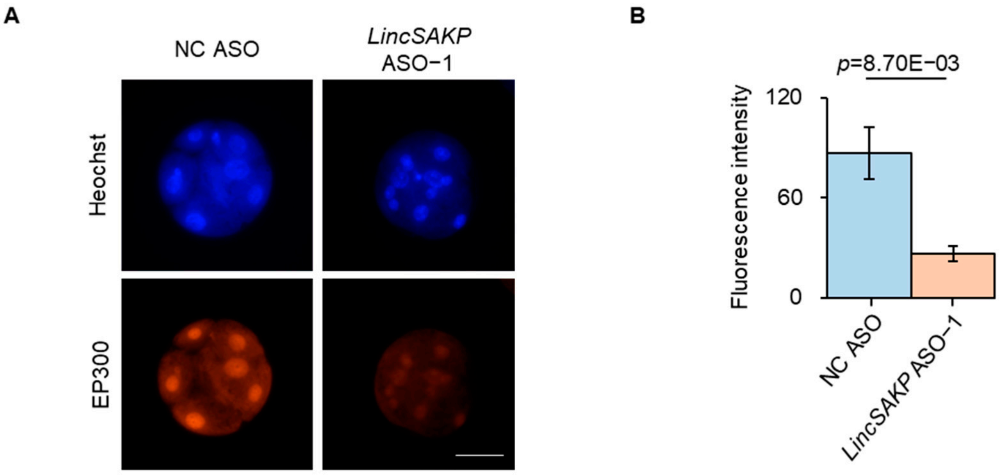

C

BL (%)					
	**NC ASO**	***EP300* ASO−1**	***EP300* ASO−2**	***EP300* ASO−3**	***EP300* ASO−4**
NC ASO	1.00E+00	1.42E−05	7.35E−06	1.03E−05	7.47E−01
EP300 ASO−1		1.00E+00	1.24E−01	7.72E−01	4.30E−05
EP300 ASO−2			1.00E+00	1.98E−01	1.48E−05
EP300 ASO−3				1.00E+00	2.92E−05
EP300 ASO−4					1.00E+00

8C arrest (%)					
	**NC ASO**	***EP300* ASO−1**	***EP300* ASO−2**	***EP300* ASO−3**	***EP300* ASO−4**
NC ASO	1.00E+00	2.82E−04	7.97E−04	1.22E−04	3.99E−01
EP300 ASO−1		1.00E+00	6.93E−01	8.11E−01	2.43E−05
EP300 ASO−2			1.00E+00	5.23E−01	7.10E−05
EP300 ASO−3				1.00E+00	1.02E−05
EP300 ASO−4					1.00E+00

4C arrest (%)					
	**NC ASO**	***EP300* ASO−1**	***EP300* ASO−2**	***EP300* ASO−3**	***EP300* ASO−4**
NC ASO	1.00E+00	8.92E−01	8.34E−01	5.44E−01	7.05E−01
EP300 ASO−1		1.00E+00	7.34E−01	6.48E−01	8.16E−01
EP300 ASO−2			1.00E+00	4.19E−01	5.59E−01
EP300 ASO−3				1.00E+00	8.17E−01
EP300 ASO−4					1.00E+00

4–8C arrest (%)					
	**NC ASO**	***EP300* ASO−1**	***EP300* ASO−2**	***EP300* ASO−3**	***EP300* ASO−4**
NC ASO	1.00E+00	4.14E−04	2.62E−03	4.21E−05	7.11E−01
EP300 ASO−1		1.00E+00	5.11E−01	5.32E−01	1.39E−04
EP300 ASO−2			1.00E+00	1.97E−01	9.38E−04
EP300 ASO−3				1.00E+00	1.32E−05
EP300 ASO−4					1.00E+00

Cleavage (%)					
	**NC ASO**	***EP300* ASO−1**	***EP300* ASO−2**	***EP300* ASO−3**	***EP300* ASO−4**
NC ASO	1.00E+00	9.18E−01	9.62E−02	8.08E−01	1.66E−01
EP300 ASO−1		1.00E+00	9.25E−02	7.38E−01	1.55E−01
EP300 ASO−2			1.00E+00	1.68E−01	7.76E−01
EP300 ASO−3				1.00E+00	2.68E−01
EP300 ASO−4					1.00E+00

Figure S5. validation of reduced EP300 protein levels following *LincSAKP* depletion and Pairwise comparison *P* values for embryonic developmental outcomes following *EP300* ASO 1–4 microinjection. **A)** Representative immunofluorescence images of EP300 in 8-cell-stage embryos following NC ASO or *LincSAKP* ASO-1 treatment. Nuclei were counterstained with Hoechst 33342. Scale bar, 100 μm. **B)** Quantification of nuclear EP300 fluorescence intensity. EP300 fluorescence was measured in all measurable nuclei within each embryo and averaged to obtain one embryo-level value; each embryo was treated as one independent statistical unit.

The NC ASO and LincSAKP ASO-1 groups each contained five embryos, distributed across three independent biological batches as 2/1/2 and 2/1/2, respectively. Thus, both treatment groups were represented in every batch. Data are shown as mean ± SEM. **C)** *P* values were obtained from pairwise contrasts based on binomial generalized linear mixed-effects models (GLMMs), with Treatment as a fixed effect and Batch as a random intercept. $P < 0.05$ was considered statistically significant.

https://doi.org/10.3390/cells15181707

A

BL (%)					
	NC ASO	LincSAKP ASO−1	EP300 ASO−1	LincSAKP ASO−1+EP300	EP300 ASO−1+LincSAKP
NC ASO	1.00E+00	4.80E−05	7.22E−05	1.57E−01	2.37E−05
LincSAKP ASO−1		1.00E+00	5.56E−01	7.07E−04	4.32E−01
EP300 ASO−1			1.00E+00	1.62E−03	8.53E−01
LincSAKP ASO−1+EP300				1.00E+00	1.02E−03
EP300 ASO−1+LincSAKP					1.00E+00

8C arrest (%)					
	NC ASO	*LincSAKP* ASO−1	*EP300* ASO−1	*LincSAKP* ASO−1+*EP300*	*EP300* ASO−1+*LincSAKP*
NC ASO	1.00E+00	9.95E−08	1.91E−07	3.02E−02	8.34E−07
LincSAKP ASO−1		1.00E+00	1.00E+00	6.81E−04	6.19E−01
EP300 ASO−1			1.00E+00	9.53E−04	6.30E−01
LincSAKP ASO−1+*EP300*				1.00E+00	3.22E−03
EP300 ASO−1+*LincSAKP*					1.00E+00

4C arrest (%)					
	NC ASO	*LincSAKP* ASO−1	*EP300* ASO−1	*LincSAKP* ASO−1+*EP300*	*EP300* ASO−1+*LincSAKP*
NC ASO	1.00E+00	6.47E−01	5.88E−01	7.26E−01	5.36E−01
LincSAKP ASO−1		1.00E+00	9.28E−01	9.11E−01	8.86E−01
EP300 ASO−1			1.00E+00	8.40E−01	9.62E−01
LincSAKP ASO−1+*EP300*				1.00E+00	7.95E−01
EP300 ASO−1+*LincSAKP*					1.00E+00

4−8C arrest (%)					
	NC ASO	*LincSAKP* ASO−1	*EP300* ASO−1	*LincSAKP* ASO−1+*EP300*	*EP300* ASO−1+*LincSAKP*
NC ASO	1.00E+00	1.28E−07	2.42E−07	4.49E−02	7.17E−07
LincSAKP ASO−1		1.00E+00	9.42E−01	7.92E−04	6.84E−01
EP300 ASO−1			1.00E+00	9.33E−04	6.40E−01
LincSAKP ASO−1+*EP300*				1.00E+00	2.75E−03
EP300 ASO−1+*LincSAKP*					1.00E+00

Cleavage (%)					
	NC ASO	*LincSAKP* ASO−1	*EP300* ASO−1	*LincSAKP* ASO−1+*EP300*	*EP300* ASO−1+*LincSAKP*
NC ASO	1.00E+00	4.79E−01	9.00E−01	4.79E−01	6.27E−01
LincSAKP ASO−1		1.00E+00	6.01E−01	9.80E−01	8.30E−01
EP300 ASO−1			1.00E+00	6.07E−01	7.50E−01
LincSAKP ASO−1+*EP300*				1.00E+00	8.44E−01
EP300 ASO−1+*LincSAKP*					1.00E+00

B

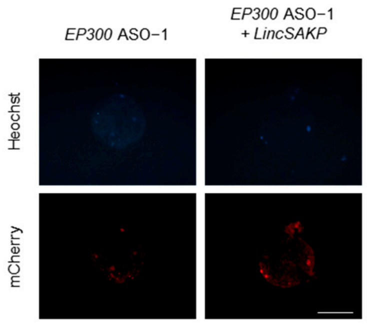

C

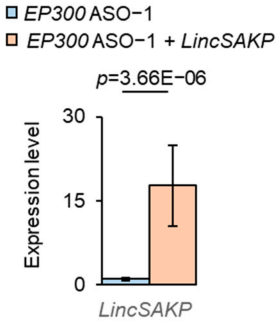

Figure S6. Pairwise comparison *P* values for embryonic developmental outcomes in the rescue experiments and validation of *LincSAKP* overexpression. **A)** Pairwise comparison *P* values for embryonic developmental outcomes following microinjection. P values were obtained from pairwise contrasts based on binomial generalized linear mixed-effects models (GLMMs), with Treatment as a fixed effect and Batch as a random intercept. P < 0.05 was considered statistically significant. **B)** RNA-FISH detection of *LincSAKP* in 8-cell-stage embryos injected with *EP300* ASO-1 alone or co-injected with *EP300* ASO-1 + *LincSAKP*. Increased *LincSAKP* signal with predominant nuclear enrichment was observed in the co-injection group. The FISH signal represents total *LincSAKP* because the probes do not distinguish endogenous from injected transcripts. Scale bar, 100 μm. Three independent biological replicates were performed. **C)** Expression levels of *LincSAKP* in

8-cell-stage embryos. After injection with *EP300* ASO-1 alone or *EP300* ASO-1 + *LincSAKP*, the RNA levels of *LincSAKP* were examined by qPCR. Error bars represent S.E.M. Approximately 50 embryos per group were used for each qPCR analysis, with three independent biological replicates. 18S rRNA served as the internal reference gene.

https://doi.org/10.3390/cells15181707

Table S1. Sequence of primers

Gene	Sequence (5'-3')		Length (bp)
LincSAKP-3′RACE-F1	forward	CATCTGGTTCAGAACTGATTGCC	
LincSAKP-3′RACE -F2	forward	GGCAGACCACTACTTACACATA	
LincSAKP-5′RACE-R1	forward	GCAATTCTCCTGTATCTCAAACTA	
LincSAKP-5′RACE-R2	forward	GGCTTTACCAGCATGTCAGTGTGAA	
LincSAKP-5′RACE-R3	forward	GCAATCAGTTCTGAACCAGATGCAT	
LincSAKP ASO-1	forward	CCAGGGTTGTACTACTAATGA	
LincSAKP ASO-2	forward	CCACAGAAATGGAGTTAAATG	
LincSAKP ASO-3	forward	GACCCACACATAAATTGTAAC	
LincSAKP ASO-4	forward	CTGCCACCAGCATCCACTACC	
EP300 ASO-1	forward	GGCCGAGAATGTGGTGGAACC	
EP300 ASO-2	forward	GACAGCGCCGAGGAGGAAGAA	
EP300 ASO-3	forward	CCACAGCCCGGTTTGAATCAG	
EP300 ASO-4	forward	GCTGGTTACCACCAGCCTGGT	
RT-*LincSAKP*	forward	CTGGTTTGAGAAATGCATCTGGTT	173
	reverse	GCTTTACCAGCATGTCAGTGTGAAT	
RT-*EP300*	forward	CGAGCGAATTGTCCACGACTAC	114
	reverse	TCCAGAACATTGGGCCAGAAAT	
RT-*ACLY*	forward	GGCCTTTCGTAGAGAGCAGG	145
	reverse	TGACCCGGGCATACTTGAAC	
RT-*EIF1A*	forward	GGTGTTCAAAGAAGATGGGCAAGAG	115
	reverse	TTTCCCTCTGATGTGACATAACCTC	
RT-*EIF3A*	forward	TAACACAGACGACGACAG	197
	reverse	CATCATCCACACCTCCTC	
RT-*PDHA1*	forward	GGATTGCTCTGGCCTGTAAG	181
	reverse	GCTCTCTCCACAGACGTTCC	
RT-*SQLE*	forward	AGGCGGTTATTCTGGTTACCG	129
	reverse	AACGGCAGTTTCCAGAGCG	
RT-*TFIIA*	forward	TCAGGAATAGAGTCAACTTCA	148
	reverse	TTACCATCACAGGCTACAA	
RT-*ZSCAN4*	forward	AGGTAAAGAATGTGGCGGCAAC	91
	reverse	GAGCAGGAAGGGCGTCTGACT	
RT-*SOX2*	forward	AACCAGAAGAACAGCCCAGAC	155
	reverse	TCCGACAAAAGTTTCCACTCG	
RT-*KLF5*	forward	GACGCGATGTGAAATGGAGAAGTA	176
	reverse	GGTGAGTGATGTCAGGGAGGAAGA	
RT-*OCT4*	forward	TCAGCCAAACGACCATCT	96
	reverse	CTTCCTCCACCCACTTCT	
RT-*NANOG*	forward	TCCTCTTCCTTCCTCCAT	108
	reverse	TCCTTCTCTGTGCTCTTC	
RT-*JUND*	forward	TCTGCCCTATTTATGTTTCTACTCGG	221
	reverse	CGAACCAAGGATTACAAACAGGAAT	
RT-*MEF2D*	forward	TCCTGTTGACGGTTACATTTGTGTAT	199
	reverse	CTGCAGCATATGTGTACAACGTGC	
RT-*TCF15*	forward	GTGTCATTTGCTGCAAGCAGGAC	108
	reverse	CCTACACGAAGAAGAGACCTGGGTT	
RT-*THRA*	forward	AAGGTGACTGACCTCCGCATGAT	116
	reverse	TGATCCTCAAAGACCTCGAGGAAG	
RT-*TP73*	forward	TTCAGTGGAACTGGAGAACACTGG	124
	reverse	GTGCTGAGGCCACTGGAGAGA	
RT-*YBX1*	forward	CCAGCAGAATTACCAGAATAGTGAGAG	163
	reverse	AGGAGGGTTGGAATACTGTGGTC	

https://doi.org/10.3390/cells15181707

RT-*ZFP57*	forward	TGGAGACCAAGTCCGACTCACAT	144
	reverse	AGCAGCGGTTGGACTGCTCTAT	
RT-*ZNF296*	forward	AGGCCATCACTGCTTTCATGGA	96
	reverse	CCTTCAGGTCATCCCGTTCTGA	
RT-*ZNF746*	forward	CCTCCACGGAAGCAGATGTAAA	93
	reverse	CTTCTAGGTCAGTAGGATGCACTGG	
RT-*KTN1*	forward	GCTGCTGGAGACACTACTGTTATTGAG	127
	reverse	CCGCTTGAAGTAATTGCTGTAACTG	
RT-*PELI2*	forward	TTGTATCTGTGGAAATACAAGCCATT	131
	reverse	CCTATTGTGTGTCCTGATGAACCAG	
RT-*18s*	forward	TCCAATGGATCCTCGCGGAA	149
	reverse	GGCTACCACATCCAAGGAAG	
RT-*U6*	forward	GCTCACTTCAGCAGCACATATAC	83
	reverse	CTTTACGAATTTTCATGGCATC	
RT-*GAPDH*	forward	TCCTGGAAGATGGTGATGGCCTTT	160
	reverse	GCAAAGTGGACATTGTCGCCATCA	
FISH-*LincSAKP*-1	forward	GTGATTCAGAGCTTACAAAAATAACTGCCTGAGT	34
	reverse	ACTCAGGCAGTTATTTTTGTAAGCTCTGAATCAC	
FISH-*LincSAKP*-2	forward	TCATTTAACTCCATTTCTGTGGGGCTCTATCCCT	34
	reverse	AGGGATAGAGCCCCACAGAAATGGAGTTAAATGA	

https://doi.org/10.3390/cells15181707

Table S2. Binomial generalized linear mixed-effects model analysis of embryonic developmental outcomes following *LincSAKP* depletion.

Endpoint	Comparison (vs NC ASO)	Mean ± SEM (%)	Model–adjusted rate (%)	95% CI for rate	Odds ratio vs NC	95% CI for OR	Raw P value	BH–FDR q value
BL (%)	*LincSAKP* ASO–1	2.3 ± 1.2	2.3	0.6–8.7	0.036	0.008–0.156	9.27E−06	1.33E−05
	LincSAKP ASO–2	5.3 ± 1.5	5.2	2.0–13.0	0.083	0.028–0.251	9.99E−06	1.33E−05
	LincSAKP ASO–3	32.6 ± 1.9	32.7	24.3–42.4	0.736	0.398–1.361	3.28E−01	3.28E−01
	LincSAKP ASO–4	7.0 ± 2.4	7.2	3.5–14.4	0.118	0.048–0.288	2.69E−06	1.08E−05
8C arrest (%)	*LincSAKP* ASO–1	38.9 ± 5.7	39.1	29.4–49.7	3.528	1.665–7.476	9.98E−04	1.33E−03
	LincSAKP ASO–2	41.8 ± 3.5	41.6	31.1–52.8	3.911	1.822–8.397	4.68E−04	1.33E−03
	LincSAKP ASO–3	16.0 ± 1.5	15.8	9.9–24.3	1.035	0.458–2.338	9.33E−01	9.33E−01
	LincSAKP ASO–4	39.2 ± 2.3	39.2	30.0–49.2	3.542	1.694–7.409	7.81E−04	1.33E−03
4C arrest (%)	*LincSAKP* ASO–1	17.2 ± 1.8	17.2	10.7–26.7	1.597	0.656–3.889	3.02E−01	3.54E−01
	LincSAKP ASO–2	18.4 ± 3.3	18.2	11.1–28.4	1.704	0.690–4.209	2.48E−01	3.54E−01
	LincSAKP ASO–3	16.8 ± 2.4	16.8	10.7–25.4	1.552	0.651–3.698	3.21E−01	3.54E−01
	LincSAKP ASO–4	16.4 ± 1.6	16.5	10.4–25.2	1.514	0.630–3.642	3.54E−01	3.54E−01
4–8C arrest (%)	*LincSAKP* ASO–1	56.1 ± 7.4	56.3	45.8–66.3	3.500	1.817–6.742	1.80E−04	2.40E−04
	LincSAKP ASO–2	60.2 ± 5.2	59.7	48.5–70.1	4.028	2.047–7.923	5.44E−05	2.18E−04
	LincSAKP ASO–3	32.8 ± 1.9	32.7	24.3–42.4	1.317	0.687–2.525	4.06E−01	4.06E−01
	LincSAKP ASO–4	55.6 ± 3.7	55.7	45.7–65.2	3.409	1.796–6.471	1.77E−04	2.40E−04
Cleavage (%)	*LincSAKP* ASO–1	83.9 ± 4.2	83.9	74.6–90.2	1.043	0.457–2.381	9.21E−01	9.21E−01
	LincSAKP ASO–2	80.8 ± 3.6	80.5	70.2–87.9	0.827	0.364–1.877	6.49E−01	9.13E−01
	LincSAKP ASO–3	86.6 ± 3.5	86.1	77.9–91.6	1.243	0.547–2.823	6.03E−01	9.13E−01
	LincSAKP ASO–4	85.3 ± 3.3	85.6	77.1–91.3	1.186	0.521–2.697	6.85E−01	9.13E−01

P values were obtained from pairwise contrasts based on binomial generalized linear mixed-effects models with Treatment as a fixed effect and Batch as a random intercept. All tests were two-sided, and $P < 0.05$ was considered statistically significant. OR, odds ratio; CI, confidence interval.

Table S3. Binomial generalized linear mixed-effects model analysis of embryonic developmental outcomes following *EP300* depletion.

Endpoint	Comparison (vs NC ASO)	Mean ± SEM (%)	Model–adjusted rate (%)	95% CI for rate	Odds ratio vs NC	95% CI for OR	Raw P value	BH–FDR q value
BL (%)	*EP300* ASO–1	7.2 ± 2.8	7.8	3.5–16.3	0.126	0.049–0.320	1.42E−05	1.90E−05
	EP300 ASO–2	2.4 ± 1.2	2.3	0.6–8.7	0.035	0.008–0.151	7.35E−06	1.90E−05
	EP300 ASO–3	6.8 ± 1.7	6.6	2.8–14.9	0.105	0.038–0.285	1.03E−05	1.90E−05
	EP300 ASO–4	37.4 ± 9.7	37.8	28.0–48.7	0.903	0.486–1.677	7.47E−01	7.47E−01
8C arrest (%)	*EP300* ASO–1	46.4 ± 1.8	46.8	35.9–57.9	3.615	1.807–7.235	2.82E−04	5.64E−04
	EP300 ASO–2	43.8 ± 1.7	43.7	33.7–54.2	3.193	1.620–6.294	7.97E−04	1.06E−03

	EP300 ASO−3	47.4 ± 7.2	48.7	37.7–59.8	3.906	1.949–7.828	1.22E−04	4.88E−04
	EP300 ASO−4	15.6 ± 2.7	14.6	8.5–24.0	0.706	0.314–1.587	3.99E−01	3.99E−01
4C arrest (%)	*EP300* ASO−1	16.8 ± 1.7	16.9	10.1–26.9	1.059	0.464–2.420	8.92E−01	8.92E−01
	EP300 ASO−2	15.3 ± 2.4	14.9	8.9–24.1	0.916	0.403–2.082	8.34E−01	8.92E−01
	EP300 ASO−3	19.7 ± 0.9	19.7	12.3–30.2	1.282	0.574–2.864	5.44E−01	8.92E−01
	EP300 ASO−4	18.8 ± 1.2	18.3	11.3–28.2	1.167	0.524–2.599	7.05E−01	8.92E−01
4–8C arrest (%)	*EP300* ASO−1	63.2 ± 3.2	63.6	52.4–73.6	3.161	1.669–5.988	4.14E−04	8.27E−04
	EP300 ASO−2	59.0 ± 3.2	58.6	48.0–68.5	2.559	1.388–4.719	2.62E−03	3.49E−03
	EP300 ASO−3	67.1 ± 7.7	68.4	57.2–77.8	3.914	2.037–7.520	4.21E−05	1.69E−04
	EP300 ASO−4	34.4 ± 3.9	32.9	23.6–43.8	0.887	0.469–1.675	7.11E−01	7.11E−01
Cleavage (%)	*EP300* ASO−1	93.3 ± 2.7	93.5	85.3–97.3	1.067	0.312–3.644	9.18E−01	9.18E−01
	EP300 ASO−2	85.6 ± 4.0	85.1	75.9–91.1	0.422	0.152–1.166	9.62E−02	3.32E−01
	EP300 ASO−3	91.2 ± 5.5	92.1	83.5–96.4	0.864	0.267–2.801	8.08E−01	9.18E−01
	EP300 ASO−4	86.6 ± 4.8	86.6	77.4–92.4	0.478	0.168–1.359	1.66E−01	3.32E−01

P values were obtained from pairwise contrasts based on binomial generalized linear mixed-effects models with Treatment as a fixed effect and Batch as a random intercept. All tests were two-sided, and $P < 0.05$ was considered statistically significant. OR, odds ratio; CI, confidence interval.

Table S4. Binomial generalized linear mixed-effects model analysis of embryonic developmental outcomes in the rescue experiments.

**Endpoint**	**Comparison (vs NC ASO)**	**Mean ± SEM (%)**	**Model−adjusted rate (%)**	**95% CI for rate**	**Odds ratio vs NC**	**95% CI for OR**	**Raw P value**	**BH−FDR q value**
BL (%)	*LincSAKP* ASO−1	3.2 ± 1.7	2.9	0.7–10.7	0.048	0.011–0.208	4.80E−05	9.60E−05
	EP300 ASO−1	4.9 ± 0.3	4.8	1.6–14.0	0.083	0.024–0.284	7.22E−05	9.62E−05
	LincSAKP ASO−1+*EP300*	28.2 ± 1.8	27.8	19.1–38.7	0.633	0.336–1.193	1.57E−01	1.57E−01
	EP300 ASO−1+*LincSAKP*	5.7 ± 1.7	5.6	2.1–13.9	0.097	0.033–0.285	2.37E−05	9.49E−05
8C arrest (%)	*LincSAKP* ASO−1	50.0 ± 1.1	50.0	38.5–61.5	8.364	3.829–18.270	9.95E−08	3.81E−07
	EP300 ASO−1	50.0 ± 5.3	50.0	37.8–62.2	8.364	3.761–18.600	1.91E−07	3.81E−07
	LincSAKP ASO−1+*EP300*	22.6 ± 1.9	22.8	14.9–33.3	2.468	1.090–5.586	3.02E−02	3.02E−02
	EP300 ASO−1+*LincSAKP*	46.0 ± 3.1	45.8	34.7–57.4	7.077	3.249–15.413	8.34E−07	1.11E−06
4C arrest (%)	*LincSAKP* ASO−1	17.6 ± 3.0	17.1	10.0–27.8	1.214	0.530–2.779	6.47E−01	7.26E−01
	EP300 ASO−1	17.6 ± 3.8	17.7	10.1–29.3	1.265	0.540–2.964	5.88E−01	7.26E−01
	LincSAKP ASO−1+*EP300*	15.7 ± 3.9	16.5	9.8–26.3	1.156	0.515–2.593	7.26E−01	7.26E−01
	EP300 ASO−1+*LincSAKP*	17.9 ± 3.1	18.1	10.8–28.7	1.293	0.574–2.913	5.36E−01	7.26E−01
4–8C arrest (%)	*LincSAKP* ASO−1	67.6 ± 2.4	67.1	55.4–77.1	6.052	3.103–11.805	1.28E−07	4.83E−07
	EP300 ASO−1	67.6 ± 2.2	67.7	55.2–78.1	6.219	3.108–12.445	2.42E−07	4.83E−07
	LincSAKP ASO−1+*EP300*	38.3 ± 5.1	39.2	29.1–50.4	1.913	1.015–3.605	4.49E−02	4.49E−02
	EP300 ASO−1+*LincSAKP*	63.9 ± 0.1	63.9	52.2–74.1	5.240	2.722–10.086	7.17E−07	9.56E−07
Cleavage (%)	*LincSAKP* ASO−1	89.9 ± 1.0	90.0	80.5–95.2	1.416	0.540–3.709	4.79E−01	8.36E−01

EP300 ASO−1	86.8 ± 2.2	87.1	76.3–93.4	1.062	0.418–2.697	9.00E−01	9.00E−01
LincSAKP ASO−1+*EP300*	89.5 ± 2.1	89.9	81.0–94.9	1.396	0.555–3.514	4.79E−01	8.36E−01
EP300 ASO−1+*LincSAKP*	88.8 ± 1.7	88.9	79.3–94.3	1.258	0.498–3.177	6.27E−01	8.36E−01

P values were obtained from pairwise contrasts based on binomial generalized linear mixed-effects models with Treatment as a fixed effect and Batch as a random intercept. All tests were two-sided, and $P < 0.05$ was considered statistically significant. OR, odds ratio; CI, confidence interval.

Disclaimer/Publisher's Note: The statements, opinions and data contained in all publications are solely those of the individual author(s) and contributor(s) and not of MDPI and/or the editor(s). MDPI and/or the editor(s) disclaim responsibility for any injury to people or property resulting from any ideas, methods, instructions or products referred to in the content.

https://doi.org/10.3390/cells15181707
